# MiR-183/-96/-182 cluster is up-regulated in most breast cancers and increases cell proliferation and migration

**DOI:** 10.1186/s13058-014-0473-z

**Published:** 2014-11-14

**Authors:** Pei Li, Cheng Sheng, Lingling Huang, Hui Zhang, Lihua Huang, Zeneng Cheng, Qubo Zhu

**Affiliations:** 1 The School of Pharmaceutical Sciences in Central South University, 172 Tongzipo Road, Yuelu District, Changsha 410013 Hunan, China; 2 The Third Xiangya Hospital of Central South University, Changsha 410013 Hunan, China

## Abstract

**Introduction:**

The *miR-183/-96/-182* cluster is a conserved polycistronic microRNA (miRNA) cluster which is highly expressed in most breast cancers. Although there are some sporadic reports which demonstrate the importance of each miRNA in this cluster in breast cancer, the biological roles of this cluster as a whole and its regulation mechanisms in breast cancer are still unclear. We compared the expression of this cluster in different cancer types, analyzed the regulation mechanism of this cluster, identified new target genes, and examined the impact of this cluster on breast cancer cells.

**Methods:**

The miRNA level was detected by LNA-based northern blot and Real-time PCR, and was also analyzed from TCGA dataset. Bioinformatics research and luciferase assay were applied to find the promoter regions and transcription factors. To investigate the biological effects of the miR-183/-96 /-182 cluster in breast cancer, we generated miR-96, miR-182 and miR-183 overexpression stable cell lines to check the overdose effects; we also used miR-Down™ antagomir for each miRNA as well as miR-183/-96 /-182 cluster sponge lentivirus to check the knockdown effects. Growth, migration, cell cycle profile and survival of these cells was then monitored by colony formation assay, MTT assay, cell wound healing assay, flow cytometry and microscopy. The target gene was validated by Real-time PCR, luciferase assay, Western blot and Phalloidin/DAPI counterstaining.

**Results:**

The *miR-183/-96/-182* cluster was highly expressed in most breast cancers, and its transcription is disordered in breast cancer. The *miR-183/-96/-182* cluster was transcribed in the same pri-miRNA and its transcription was regulated by *ZEB1* and *HSF2*. It increased breast cell growth by promoting more rapid completion of mitosis, promoted cell migration and was essential for cell survival. *MiR-183* targeted the *RAB21* mRNA directly in breast cancer.

**Conclusion:**

The *miR-183/-96/-182* cluster is up-regulated in most breast cancer. It functions as an oncogene in breast cancer as it increases cell proliferation and migration.

**Electronic supplementary material:**

The online version of this article (doi:10.1186/s13058-014-0473-z) contains supplementary material, which is available to authorized users.

## Introduction

Breast cancer is a family of diseases that involve unregulated breast epithelial cell growth and division, which is caused by many different carcinogenic factors. The exact cause of breast cancer is unclear. Many risk factors may increase the chance of having breast cancer, such as endocrine disorders, genetic mutations and declines in immune function. However, unregulated mammary epithelial cell proliferation and apoptosis, which are caused by an accumulation of gene mutations and by dysregulated gene expression, is the essential reason for breast cancer. As numerous genes are predicted to be regulated by microRNA (miRNA), mammary tumorigenesis and metastasis is likely to be regulated by several tissue-specific miRNAs.

The *miR-183/-96/-182* cluster is a highly conserved polycistronic miRNA cluster which was first identified by Dr Xu in sensory organs [1]. Members of this cluster are located within a 5-kb region on human chromosome 7q32.2 and are transcribed in the same direction from telomere to centromere. Previous studies showed that the *miR-183/-96/-182* cluster is abnormally expressed in a variety of tumors and is directly involved in human cancers. But the role of this miRNA cluster in tumors is still unclear. It may function as an oncogene or tumor suppressor gene, depending on the type, location and stage of the cancer. We summarize its reported functions in cancers and its target genes in Table 1.

**Table 1:** **Role of miR-183/-96/-182 in cancer based on recent publications within the last five years**

miRNA	Oncogene/tumor suppressor	Cancer type	Function	Target genes	Reference
miR-96	Oncogene	Hepatocellular carcinoma	Increases proliferation and colony formation	*FOXO1, FOXO3a*	[2]
miR-96	Oncogene	Prostate cancer	Inhibits zinc uptake	*ZIP1, ZIP3, ZIP7, ZIP9, ZnT1, ZnT7*	[3]
miR-182					
miR-183					
miR-96	Oncogene	Medullo-blastoma	Inhibits apoptosis, destroys DNA repair, promotes cell migration	*See reference*	[4]
miR-182					
miR-183					
miR-96	Oncogene	Breast cancer	Induces proliferation	*FOXO3a*	[5]
miR-96	Oncogene	Breast cancer	Increases cell number	*FOXO1*	[6]
miR-182					
miR-182	Oncogene	Glioma	Promotes glioma cell aggression	*CYLD*	[7]
miR-182	Oncogene	Melanoma	Promotes cell migration and survival	*FOXO3*	[8]
miR-183	Oncogene	Synovial sarcoma	Promotes tumor cell migration	*EGR1*	[9]
				*PTEN*	
miR-183	Oncogene	Hepatocellular carcinoma	Iinhibits TGF-beta1-induced apoptosis	*PDCD4*	[10]
miR-96	Tumor suppressor	Pancreatic cancer	Decreases cell invasion, migration and tumor growth	*KRAS*	[11]
miR-183	Tumor suppressor	Breast cancer	Inhibits migration	*Ezrin*	[12]
miR-183	Tumor suppressor	Osteosarcoma	Inhibits migration and invasion	*Ezrin*	[13]
miR-182	Tumor suppressor	Lung cancer	Inhibits cancer cell proliferation	*RGS17*	[14]

The *miR-183/-96/-182* cluster has not yet been extensively studied in breast cancer. Forkhead box O (*FOXO*) proteins, which are a family of tumor suppressor transcription factors involved in cell growth, proliferation, differentiation, and longevity, are the main targets for this cluster in breast cancer. Both *FOXO1* and *FOXO3a* are regulated by *miR-96* and *miR-182* [5],[6]. It seems that this miRNA cluster functions as onco-microRNA in breast cancer. However, in 2010, *Lowery et al.* reported that *miR-183* inhibits cell migration in breast cancer by repressing *Ezrin*, which plays a key role in cell-surface structure adhesion, migration, and organization [12]. These conflicting results may be ascribed to two reasons. One possibility is that these three miRNAs are transcribed or processed in different way and they function separately and differently; the other possibility is that this cluster plays different roles in different breast cancer types. In fact, the level of *miR-183* was lower in estrogen receptor (*ER)*-positive breast tumors compared to *ER*-negative tumors, and higher in human epidermal growth factor receptor-2 (*HER2)/neu-*receptor-positive tumors compared to *HER2/neu-*receptor-negative tumors [12], suggesting the roles of *miR-183* in different breast cancer cells are different.

Recently, attention has focused on the target genes of these miRNAs; however, little is known about the regulation mechanism of the miRNA cluster itself. Most miRNA genes are transcribed by RNA polymerase II [15], which means miRNA biogenesis is controlled elaborately through various regulatory pathways just as protein-coding mRNAs. Chromatin structure analysis, genomic and RNA sequence analysis and RNA polymerase II chromatin immuneprecipitation assays have been applied to predict the transcription start site (TSS) and promoter region of miRNAs [16]-[19], but few results have been confirmed by experiments. The Ozsolak [16], Wang [18], and Chien [19] laboratories predicted that the TSS of *miR-183/-96/-182* was 5068 bp, 5200 bp and 5207 bp upstream of the *miR-183* precursor, respectively. However, the promoter region of *miR-183/-96/-182* and the transcription regulators remain unknown.

Here, we investigated the function of the *miR-183/-96/-182* cluster in breast cancer. We found that the *miR-183/-96/-182* cluster was highly expressed in most breast cancers. These three miRNAs were transcribed in the same pri-miRNA and this miRNA cluster was regulated by *HSF2* and *ZEB1*. We also demonstrated that the m*iR-183/-96/-182* cluster functioned as an onco-miRNA in breast cancer. Overexpression of the *miR-183/-96/-182* cluster increased the cell proliferation rate and promoted cell migration while inhibition of the *miR-183/-96/-182* cluster decreased cell growth rate, and even induced cell death. *MiR-183* targeted *RAB21* directly in breast cancer and accumulated nucleus number aberration cells. Our results suggested that the *miR-183/-96/-182* cluster plays an important role in tumorigenesis and in the migration of breast cancer cells.

## Methods

### Clinical cancer samples and cell lines

All cancer samples were obtained from the Affiliated Tumor Hospital of XiangYa Medical School of Central South University, and stored at -80°C until analyzed. All experiments were conducted in accordance with the Declaration of Helsinki and were approved by the Xiangya Hospital Medical Ethics Committee in Central South University.

Breast cancer cell lines MCF-7,MDA-MB-231,SK-BR-3,T47D, ZR-75-1, MCF-10A and human embryonic kidney cell HEK-293 were used in the study. MCF-7 and MDA-MB-231 were obtained from NeuronBiotech (Shanghai, China). SK-BR-3, T47D, ZR-75-1 and MCF-10A were obtained from Dingguo, Co. (Beijing, China). HEK-293 was obtained from Xiangya experiment center (Changsha, China). All the cells were cultured in complete DMEM high glucose medium (Hyclone, Logan, UT, USA) supplemented with 10% FBS (Hyclone) and 1% penicillin and streptomycin sulfate (Solarbia, Co., Beijing, China). Cells were incubated at 37°C with 5% CO_2_ and medium was changed every 2 or 3 days.

### Virion and cell line constructions

To establish the miRNA overexpression cell lines, partial *mir-96*, *mir-182* and *mir-183* pri-microRNA sequences flanked by EcoRI and AgeI restriction sites were inserted into the CMV promoter of lentivirus infectious virions pLKD-CMV-G&PR-U6-shRNA (Hpcoo3) (Additional file 1: Figure S1A). MCF-7 or T47D cells were infected with these viruses and selected under the pressure of 1 μg/ml puromycin (Invitrogen, San Diego, CA, USA). The green fluorescent protein (GFP) signal of the infected cells was detected under microscope (Additional file 1: Figure S1B), and the expression of the *miR-183/-96/-182* cluster in each cell line was measured by reverse transcription (RT)-PCR (Additional file 1: Figure S1C).

To disrupt the activity of the *miR-183/-96/-182* cluster, we generated *miR-183/-96/-182* cluster sponge lentivirus virion. Basically, 10 copies each of complementary sequences to *miR-183*, *miR-96* and *miR-182*, each with mismatches at positions 9 to 12 for improved stability [20],[21], were introduced into the pLOV-CMV-eGFP-EF1a-PuroR lentivirus infective virion (Additional file 2: Figure S2). A moderate multiplicity of infection (MOI) of 1 was used for transduction. The infection efficiency and cell morphology were monitored under microscope every day. After 3 days of transduction, cells were collected for cell cycle analysis and RNAs were collected for real-time PCR.

To research the function of transcription factors, the coding sequences of *HSF2* and *ZEB1* flanked by XhoI and KpnI restriction sites were inserted into vector GV219. The plasmids were transfected into MCF-7 cells and the cells were selected with a culture medium containing 600 μg/ml G418-Geneticin (GenView, Galveston, TX, USA) for 2 months.

### LNA-based Northern Blotting

Total RNAs were extracted from cancer samples with the mirVanaTM miR isolation kit and 10 μg of total RNA was used for each assay. All procedures followed manufacturer's instructions for the miRCURY LNA™ microRNA detection probes (Exiqon, Woburn, MA, USA). After fractionation by electrophoresis on a denaturing 12% polyacrylamide gel containing 8 M urea, RNAs were transferred to Nytran N membrane (Amersham Biosciences, Piscataway, NJ, USA) and fixed by UV crosslinking. Blots were prehybridized for 1 h at 45°C in PerfectHyb™ Plus Hybridization Buffer (Sigma, St Louis, MO, USA) and hybridized overnight at 45°C in hybridization buffer containing 0.1 nM probe, then washed twice for 30 minutes at 65°C in 0.1SSC/0.1% SDS. As the probes were 5'-DIG labeled, we detected the signal by PhototopeR-Star Kit (New England BioLabs Inc, Ipswich, MA, USA), and the densities were quantified by the Image J program. Because the *miR-183, miR-96* and *miR-182* sequences are similar, we tested the probe specificities before doing the experiments (Additional file 3: Figure S3). Mimic oligonucleotides were designed based on miRNA sequences registered in the miRBase Sequence Database (see Additional file 4: Table S1).

### RT-PCR and real-time PCR

For mRNA RT-PCR and real-time PCR, total RNAs were extracted from cancer samples or cultured cells with Trigol (Dingguo, Co.) reagent. Primer sets were designed within the exon junction areas listed in Additional file 4: Table S2. For miRNA real-time PCR, miRNAs were extracted from cells using a mirVana miRNA isolation kit (Ambion, Austin, TX, USA). All primers, including the YRBIO™ miRNA qPCR Detection primer sets and U6 snRNA PCR primer set were purchased from Yingrun Biotechnology (Changsha, China).

In brief, mRNA and miRNA were reverse-transcribed with an M-MLV First Strand kit (Invitrogen). Then 50 ng cDNA was mixed with All-in-one™ qPCR Mix (Genecopoeia, Rockville, MD, USA) and the target gene primer set (final concentration: 1 μM for each primer) to produce a 20-μl reaction mixture. All real-time PCR experiments were carried out with an ABI Step One Plus Real-time PCR System (Applied Biosystems, Carlsbad, CA, USA). All real-time PCR reactions were done in triplicates, and the average ΔCT (Δ cycle threshold) for the triplicates was used in subsequent analysis.

### Plasmid, miR-Down™ antagomir and transfection

Large-scale plasmids were extracted by PureYield™ Plasmid Midiprep System (Promega, Madison, WI, USA), and small-scale plasmids were extracted by Mini DNA purification kit (Dingguo). Chemically modified antisense oligonucleotides (miR-Down™ antagomir, GenePharm Co. Ltd, Shanghai, China) were used to inhibit *miR-96*, *miR-182* and *miR-183* expression. A scrambled oligonucleotide was used as control. Plasmid and miR-Down™ antagomir transfections were conducted with Lipofectamine™ 2000 reagent (Invitrogen).

### Luciferase reporter assays

For promoter analysis, promoter region sequences or their mutants flanked by XhoI and KpnI restriction sites were inserted into the upstream region of luciferase reporter gene in pGL3-Basic vector (Promega). MCF-7 cells were transfected with 200 ng reporter construct and 1 μg GV219 vector with or without transcription factor sequence. Also, 40 ng of pRL*-*CMV-Renilla plasmid was transfected as an internal control*.*

For target analysis, 33 bp of *RAB21* 3'-UTRs including the seed sequence were flanked by XbaI and FseI restriction sites and inserted between the Luciferase coding sequence and SV40 polyadenylation element in pGL3-Promoter vector (Promega). HEK-293 cells were transfected with 200 ng reporter construct and 1 μg Hpcoo3 vector with or without partial pri-microRNA sequence of *miR-183/-96/-182* cluster. Also, 40 ng of pRL*-*CMV-Renilla plasmid was transfected as an internal control*.*

The luciferase reporter assays (Promega) were performed 48h after transfection, and luciferase activity was determined with a GloMax 20/20 Luminometer (Promega). Relative luciferase activities were calculated as ratios of firefly to renilla luciferase activities.

### Assays: 3-(4, 5-dimethyl-2-thiazolyl)-2, 5-diphenyl-2H-tetrazolium bromide (MTT)

Cells were seeded on 96-well plates (5 × 10^3^ cells per well) and incubated for 24 h in 0.2 ml medium. After reaction with 20 μl 5 mg/ml sterile MTT (Sigma) for 4 h at 37°C, culture media was removed and 150 μl of dimethyl sulphoxide (DMSO) was added. The absorbance was measured with the ELISA reader (BioTek, Vermont, VT, USA) at 490 nm and 540 nm and the reactions were performed in triplicates.

### Cell wound-healing assays

Cells were seeded on 6-well plates (5 × 10^5^ cells per well) and incubated for 24 h. Adherent cell monolayers were scratched with a 10-μl pipette tip and cultured in 2 ml DMEM high-glucose medium without FBS or antibiotics. Cell migration was monitored under microscopy later.

### Colony formation assays

The culture dish was covered by 2 ml bottom gel (0.5% basic agar in RPMI medium 1640 (Invitrogen) supplemented with 10% FBS and 1% penicillin/streptomycin) and 1.5 ml top gel (0.7% agar in RPMI-1640 medium supplemented with 10% FBS and 1% penicillin/streptomycin) mixed with 10,000 cells. Cells were incubated for 16 days and the colonies were stained with 0.5ml 0.005% crystal violet overnight followed by washing with PBS (Hyclone) three times. The pictures of cell colonies were taken by a digital camera.

### Cell cycle analysis

Cells were digested with 0.05% trypsin (Thermo Scientific, Logan, UT, USA) for 2 minutes to dissociate them from the plates. After fixation in 70% pre-chilled (−20°C) ethanol in PBS at 4°C overnight, cells were treated with 10 μg/ml of RNase (Auragene, Co., Shenzhen, China) in PBS at 37°C for 2 h and stained with 50 μg/ml of propidium iodide (PI) (Sigma) for 5 minutes. Flow cytometry was conducted on a BD FACSCalibur flow cytometer (BD Biosciences, Franklin, IN, USA) and data were analyzed by ModFit LT software.

### Western blotting

Total proteins were lysed in RIPA buffer (150 mM NaCl, 0.1% SDS, 0.5% sodium deoxycholate, 1% NP-40 and 50 mM Tris-HCl, pH 7.6) with a proteinase inhibitor cocktail (Roche, Mannheim, Germany). After separation by 15% polyacrylamide gels and transfer to 0.45 μM membrane (Millipore, Billerica, MA, USA), proteins were detected by *anti-RAB21* (Abcam, HongKong, China) and *anti-β-tubulin* (Sigma) antibodies.

### Phalloidin and 4',6-diamidino-2-phenylindole (DAPI) staining

For imaging of fixed cells, cells were seeded on acid-washed, glass coverslips coated with 5 μg/ml of collagen. Cells were then fixed with 3.7% paraformaldehyde in PBS permeabilized with 0.2% Triton X-100 in PBS for 15 minutes. Then we co-stained the cells with fluorescein isothiocyanate (FITC)-conjugated phalloidin (Beyotime, Shangai, China) to detect the F-actin, and with DAPI (Invitrogen) to detect the nuclear. Coverslips were mounted with Microscopy Aquatex® mounting medium (Merck, Darmstadt, Germany), and then detected under the Leica Tcs-sp5-II confocal microscope (Leica, Wetzlar, Germany).

### Statistical analysis

Data were expressed as means ± SD, and the statistical software SPSS 11.5 (IBM, Armonk, NY, USA) was used for analysis of variance (ANOVA) and analysis using Student's *t*-test. Statistical probability (*P*) in tables, figures, and figure legends are expressed as follows: *P <0.05, **P <0.01, *** *P* <0.001.

## Results

### The miR-183/-96/-182 cluster was highly expressed in most breast cancers

**Figure 1:**
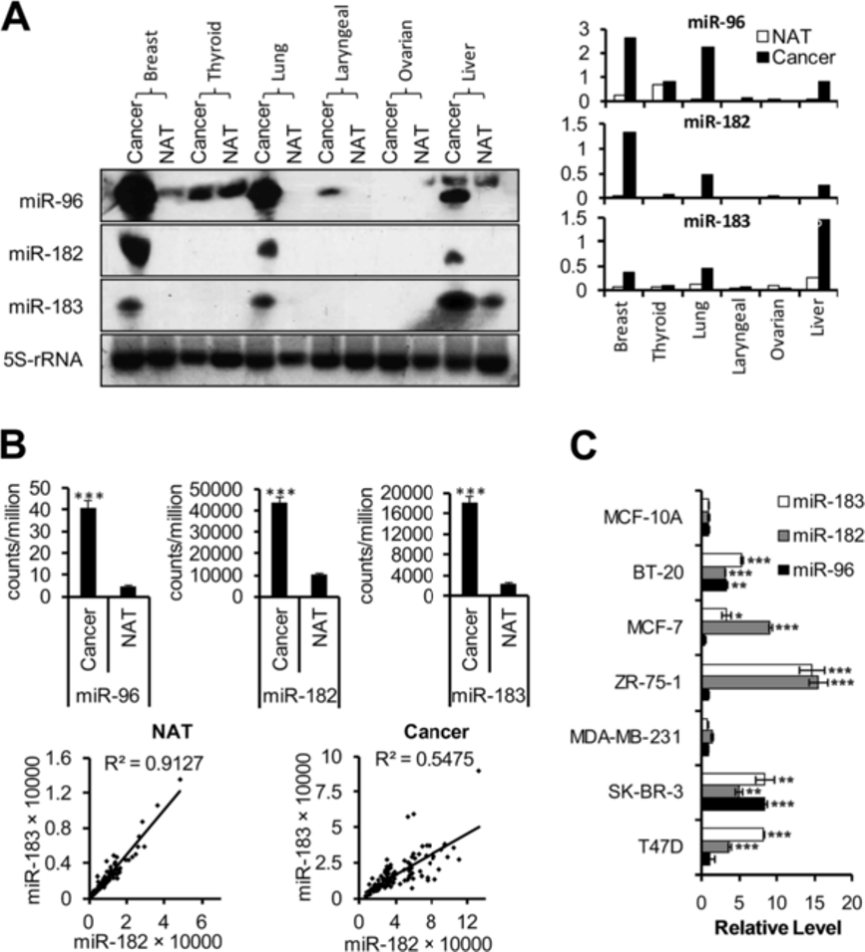
**MiR-183/-96/-182 cluster is highly expressed in breast cancer cells. (A)** Detection of *miR-183/-96/-182* cluster miRNAs by LNA-based northern blot in different cancer samples and their normal adjacent tissues (NAT). Left panel shows the northern blot analysis of *miR-183/-96/-182* cluster miRNAs; 5S-r RNA was used as an internal control. Left panel is the quantification of selected miRNAs by the Image J program. The results were normalized to the 5S-r RNA. **(B)** Statistical analysis of miRNA expression data in breast invasive carcinoma from the TCGA dataset: upper panel compares the miRNA expression levels between tumor samples and their matched normal samples; lower panel analyzes the correlation between *miR-182* and *miR-183* levels in normal and tumor samples. Error bars indicate SD (n = 102). **(C)** Quantification of the *miR-183/-96/-182* cluster miRNAs by real-time PCR in different breast cancer cell lines. MCF-10A cell was used as control. U6 snRNA was used as internal control. Error bars indicate SD (n = 3).

Six different tumors and their normal adjacent tissues (NAT) were collected from the Hunan Tumor Hospital. Breast cancer and liver cancer tumors were available from two patients, and other types of cancer were from one patient. The miRNAs were detected by LNA-based northern blot. We found that *miR-96*, *miR-182* and *miR-183* expression levels were dramatically higher in tumors compared to the normal adjacent tissues in breast, lung and liver cancers. *MiR-96* was also expressed in thyroid and larynx cancers, but the expression differences between tumors and their normal adjacent tissues were not obvious. The expressions of these three miRNAs were undetectable in other carcinoma tissues (Figure 1A). We then performed an analysis of miRNA expression data detected by either IlluminaGA_miRNASeq or IlluminaHiseq_miRNASeq in breast invasive carcinoma from the TCGA dataset. From 102 matched pairs of samples (Additional file 5), we found the expression levels of *miR-96*, *miR-182* and *miR-183* in tumor samples were increased 8.4 (± 1.1)-fold, 4.2 ± (1.1)-fold and 7.5 ± (1.1)-fold respectively compared to the matched normal samples (Figure 1B, upper panel). Another interesting phenomenon was that the expression levels of *miR-183* and *miR-182* were highly correlated in normal samples (*R* ^2^ = 0.9127), but the correlation dropped dramatically in tumor samples (*R* ^2^ = 0.5475), which indicated that the transcription pattern was changed in breast cancer (Figure 1B, lower panel).

Because breast cancer is a heterogeneous disease comprising different subtypes that vary significantly with regard to clinical features and molecular markers, we compared the miRNAs expression levels in different breast cancers based on their clinical features, surface markers and clinical stages. From 990 samples (Additional file 6), we found the expressions of *miR-96* and *miR-183* were lower in lobular carcinoma than in ductal carcinoma and other types of carcinoma, but the expression of *miR-182* was not correlated with the clinical features (Table 2). The levels of *miR-96* and *miR-183* were also lower in ER+ and PR+ cancers than in ER− and PR− cancers, but *miR-182* was almost the same, even slightly higher in ER+ cancers. We did not find any correlation between the *miR-183/-96/-182* cluster level and the HER2/neu receptor (Table 3). The expression of *miR-183/-96/-182* cluster was not correlated with clinical stages, as all the three miRNAs remained the same in all clinical stages (Table 4). To divide the breast cancer samples into different subtypes, the following surface markers were used: luminal A (ER+ and/or PR+, HER2−), luminal B (ER+ and/or PR+, HER2+), basal-like (ER-, PR-, HER2-), HER2-enriched (ER-, PR-, HER2+) [22]. We found *miR-96* and *miR-183* levels were higher in HER2-enriched breast cancers than other types. In basal-like breast cancers, *miR-182* was lower but *miR-183* was higher comparing to other types of breast cancer (Table 5). All these data indicated that although *miR-183/-96/-182* cluster was up-regulated in most breast cancers, its expression pattern was slightly different in different breast cancer subtypes.

**Table 2:** **Correlation between miRNA levels and clinical features**

	miR-96 (per million)	miR-182 (per million)	miR-183 (per million)
**Ductal (n = 734)**	43.0 ± 34.4	48741.7 ± 33619.5	20501.7 ± 15436.4
**Lobular (n = 163)**	36.6 ± 28.8^*^	51136.1 ± 37893.2	14014.1 ± 9845.4^***^
**Mixed (n = 28)**	37.6 ± 26.1	44265.7 ± 22469.4	15781.5 ± 10831.5
**Other (n = 63)**	49.1 ± 41.2	50700.0 ± 38025.3	19988.6 ± 17839.2

The expression of each miRNA in the *miR-183/-96/182* cluster in different breast cancer subtypes is based on their clinical features: Patient number is indicated in the first column. Data are presented as mean ± SD. Statistical probability (*P*) was expressed as *P<0.05, ***P<0.001.

**Table 3:** **Correlation between miRNA levels and surface markers**

	miR-96 (per million)	miR-182 (per million)	miR-183 (per million)
**ER− (n = 185)**	46.9 ± 37.1	42764.4 ± 29615.6	23463.0 ± 18606.7
**ER + (n = 643)**	40.5 ± 32.2^*^	49378.5 ± 32671.3^*^	17756.8 ± 12451.0^***^
**PR+ (n = 265)**	45.6 ± 34.7	45513.2 ± 32873.7	22037.2 ± 16212.9
**PR + (n = 561)**	40.2 ± 32.8^*^	49013.4 ± 31716.4	17628.8 ± 12993.5^***^
**HER2− (n = 512)**	40.7 ± 34.5	47943.7 ± 32825.1	18532.7 ± 14331.1
**HER2+ (n = 144)**	41.8 ± 32.5	44664.8 ± 28806.9	19404.0 ± 14591.3

The expression of each miRNA in the *miR-183/-96/182* cluster in different breast cancer subtypes based on their surface markers: Patient number is indicated in the first column. Data are presented as mean ± SD. Statistical probability (*P*) was expressed as *P<0.05, ***P<0.001. ER, estrogen receptor; PR, progesterone receptor; HER, human epidermal growth factor receptor.

**Table 4:** **Correlation between miRNA levels and clinical stages**

	miR-96 (per million)	miR-182 (per million)	miR-183 (per million)
**Stage I (n = 168)**	39.6 ± 31.5	48001.8 ± 29113.8	17783.2 ± 14764.5
**Stage II (n = 565)**	44.1 ± 35.7	49987.5 ± 37649.4	19919.8 ± 15730.5
**Stage III (n = 222)**	38.6 ± 30.2	47865.8 ± 29685.2	18164.0 ± 11961.5
**Stage IV and X (n = 33)**	44.2 ± 34.2	47104.5 ± 29358.6	22262.4 ± 17792.5

The expression of each miRNA in the *miR-183/-96/182* cluster in different breast cancer subtypes based on their clinical stages: Patient number is indicated in the first column. Data are presented as mean ± SD.

**Table 5:** **miRNA levels in different molecular subtypes of breast cancer**

	miR-96 (per million)	miR-182 (per million)	miR-183 (per million)
**HER2-enriched (n = 34)**	55.0 ± 46.9^*^	49308.2 ± 31807.6	24494.0 ± 16871.2^*^
**Basal (n = 105)**	42.3 ± 30.6	38971.0 ± 24170.6^**^	21991.9 ± 16491.6^*^
**Luminal A (n = 406)**	40.4 ± 35.5	50347.2 ± 34351.2	17668.1 ± 13604.2
**Luminal B (n = 109)**	38.0 ± 25.5	43312.6 ± 27928.1	17779.5 ± 13563.3

The expression of each miRNA in *miR-183/-96/182* cluster in different molecular subtypes of breast cancer: Patient number is indicated in the first column. Data are presented as mean ± SD. The following markers were used to determine breast cancer subtypes: luminal A (estrogen receptor (ER)+ and/or progesterone receptor (PR)+, human epidermal growth factor (HER)2−), luminal B (ER + and/or PR+, HER2+), basal-like (ER-, PR- , HER2−), HER2-enriched (ER-, PR-, HER2+). Statistical probability (*P*) was expressed as *P<0.05, **P<0.01.

To confirm our findings, we also compared the miRNAs levels in different breast cancer cell lines based on their ER, PR and HER2/neu receptor status. T47D (ER+/PR+/HER2−), SK-BR-3 (ER-/PR-/HER2+), MD-MBA-231 (ER-/R−/HER2−), ZR-75-1 (ER+/PR+/HER2+), BT-20 (ER-/PR-/HER2−) and MCF-7 (ER+/PR+/HER2-) cell lines were tested in this study and normal human mammary epithelial cell line (MCF-10A) were used as a control. We found that, relative to MCF-10A cell expression levels, *miR-96* was only up-regulated in SK-BR-3 and BT-20 cells; *miR-18*2 and *miR-183* were up-regulated in most of the breast cancer cell lines except MD-MBA-231; none of the miRNAs in the *miR-183/-96/-182* cluster was increased in MD-MBA-231 cell line (Figure 1C). Our data were similar to those reported by *Riaz et al.* [23], who also found that the highest expression of *miR-96* was SK-BR-3 and the lowest expression of all these three miRNAs was MD-MBA-231 among these six breast cancer cell lines. We chose MCF-7 and T47D cells for further studies because their *miR-183/-96/-182* clusters were highly expressed and they were easy to culture.

### MiR-183/-96/-182 cluster was transcribed in the same pri-miRNA and was regulated by ZEB1 and HSF2

**Figure 2:**
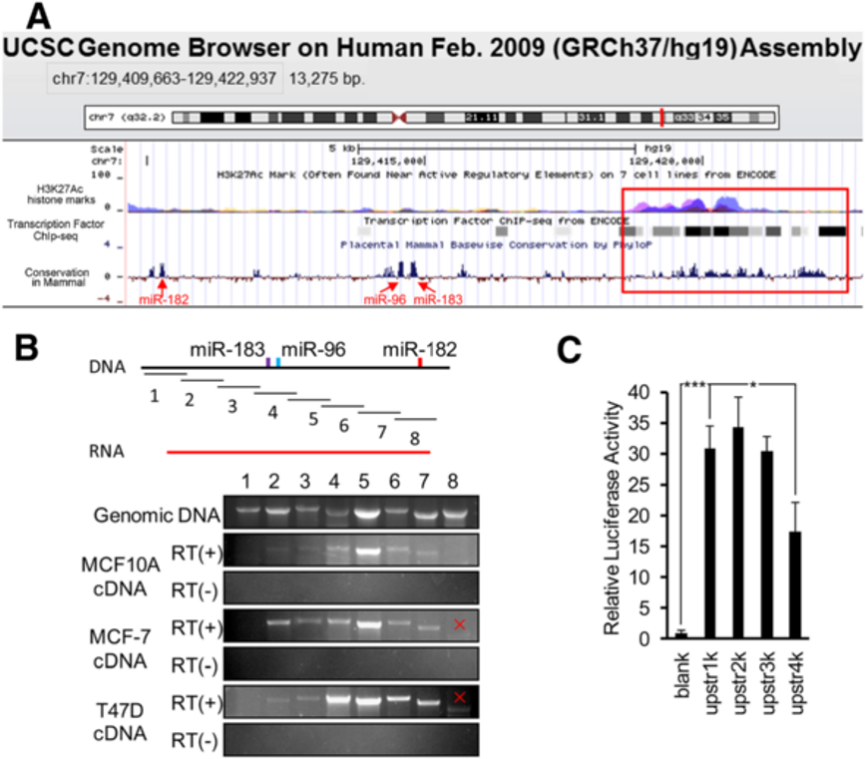
**Analysis of the miR-183/-96/-182 cluster promoter region. (A)** ENCODE project analysis of the upstream sequence of the miR-183/-96/-182 cluster: sequences in the red box represent the region from 5054 bp to 9324 bp upstream of the human miR-183 precursor that is highly conserved and enriched for 3K27Ac histone marks. **(B)** Fragmental reverse transcription (RT)-PCR demonstrated that the *miR-183/-96/-182* cluster was transcribed in the same pri-miRNA: upper panel shows a schematic representation of the location of RT-PCR fragments and the *miR-183/-96/-182* cluster in chromosome; lower panel shows the RT-PCR results of MCF-10A, MCF-7 and T47D cell cDNAs. Genomic DNA of MCF-7 cell was used as a positive control to check the efficiency of primer pairs; RNA sample, which did not undergo the reverse transcription reaction, was used as a negative control. **(C)** Luciferase assay indicated that most active regulatory elements were located within 1 kb from upstream of the TSS region of *miR-183/-96/-182* cluster. All luciferase activities were normalized to those obtained with the pGL3-Basic vector alone. Error bars represent SD (n = 4).

To study the regulation mechanism of the *miR-183/-96/-182* cluster itself, we first analyzed the upstream sequence of the *miR-183/-96/-182* cluster through the ENCODE project. We found a highly conserved region from 5054 bp to 9324 bp upstream of the human *miR-183* precursor (Figure 2A, red box). The ENCODE project displayed the acetylation of histone H3 and the transcription factor chromatin immunoprecipitation (Chip) data to find the active regulatory elements. H3K27Ac histone marks were enriched in this region, which demonstrates that this region contains active regulatory elements. Transcription factor Chip data also showed that this region was easily pulled down with transcription factors. Altogether the information suggested that the promoter region and TSS of the *miR-183/-96/-182* cluster is in this region.

Then, to check whether *miR-183*, *miR-96* and *miR-182* were transcribed in the same pri-miRNA or separately, we designed a series of primer pairs (Additional file 4: Table S3) to determine whether the corresponding regions of DNA were transcribed. Each primer pair spanned about 1600 bp and all the primer pairs divided the genomic DNA surrounding the *miR-183/-96/-182* cluster (5352 bp upstream to 5893 downstream of human *miR-183* precursor) into eight regions. From 5'-end to 3'-end, they were named Seq#1, Seq#2 … Seq#8 (their relative locations are showed in Figure 2B, upper panel). Total RNAs were extracted from MCF-7, T47D and MCF10A cell lines. MCF-7 genomic DNA was used as a positive control to check the efficiency of primer pairs. RT-PCR data showed that RNA were correctly transcribed from Seq#2 to Seq#7 (Seq#8 was a non-specific band because the size is incorrect) (Figure 2B). This data indicated that *miR-183*, *miR-96* and *miR-182* were transcribed in the same pri-miRNA and the start site of this pri-microRNA was 5352 bp to 3991 bp upstream of the *miR-183* precursor, and the transcript termination site was 289 bp to 1352 bp downstream of the *miR-182* precursor. Several papers also predicted that the TSS of *miR-183/-96/-182* was between 5068 bp and 5207 bp upstream of human *miR-183* precursor [16],[18],[19]. We could not tell whether the transcription pattern was changed in cancer cells from this experiment because the PCR method is not linear.

Next we sought to determine how this pri-miRNA was regulated. To find the promoter region, we generated luciferase reporters with 1 kb, 2 kb, 3 kb and 4 kb DNA fragments within the conserved region (4263 bp to 8533 bp upstream of the mouse *miR-183* precursor, corresponding to 5054 bp to 9324 bp upstream of the human *miR-183* precursor. Figure 2A, red box), named upstream 1 kb, upstream 2 kb, upstream 3 kb and upstream 4 kb respectively. These luciferase assay results showed that the upstream 1 kb, upstream 2 kb and upstream 3 kb fragments increased luciferase activity approximately 30-fold compared with the empty vector. No significant difference was detected among upstream 1 kb, upstream 2 kb and upstream 3 kb. Upstream 4 kb increased luciferase activity 17-fold compared with the empty vector, which was much lower than the other three reporters (Figure 2C). These data demonstrate that most active regulatory elements were located within 1 kb from the upstream of TSS region, and some repression elements were located between 3 kb and 4 kb from upstream of the TSS region.

**Figure 3:**
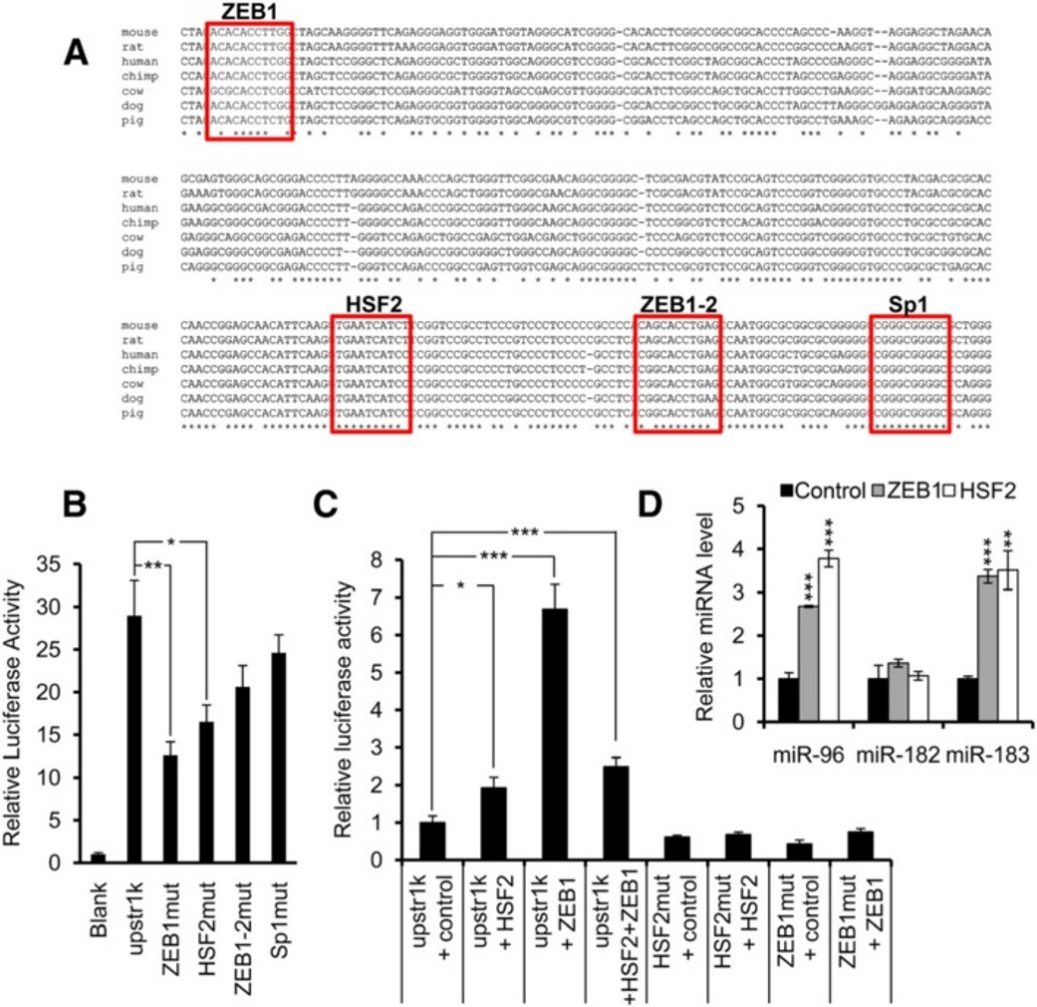
**Identification of the transcription factors regulating the miR-183/-96/-182 cluster. (A)** Phylogenetic analysis demonstrated that there were four conserved transcription factor binding sites located within the 1 kb region upstream of the TSS of the *miR-183/-96/-182* cluster in vertebrates. **(B)** Luciferase activities were decreased after mutation of the first *ZEB1* and *HSF2* transcription factor binding sites. All luciferase activities were normalized to those obtained with the pGL3-Basic vector alone. Error bars represent SD (n = 3). **(C)** Transfection of *ZEB1* and *HSF2* transcription factors could elevate the luciferase activity of native upstream 1 kb luciferase reporter but not its mutants. All luciferase activities were normalized to those obtained with the native upstream 1 kb alone. Error bars represent standard deviation (n = 3). **(D)** Real-time PCR showing that *miR-96* and *miR-183* levels were increased in *ZEB1* and *HSF2* overexpressing MCF-7 cell lines. U6 snRNA was used as internal control. Error bars represent SD (n = 3).

To find the transcription factors regulating the *miR-183/-96/-182* cluster, we used the online bioinformatics tools TFSEARCH to predict the transcription factor binding sites within 1 kb upstream from the TSS region of the *miR-183/-96/-182* cluster. Four DNA sequences were predicted to be recognized by *ZEB1*, *HSF2*, *ZEB1* and *Sp1* respectively (Figure 3A). We mutated the candidate transcription factor binding sites and performed the luciferase assay again. The luciferase activities of the *HSF2* and the first *ZEB1* mutant were significantly lower than upstream 1 kb (by about 50%), which suggested that these two sites were indeed transcription factor binding sites and that *HSF2* and *ZEB1* were two important transcription factors in cluster transcriptional regulation (Figure 3B). Therefore, we cloned *HSF2* and *ZEB1* into the GV219 vector and co-transfected the transcription factors and the native or mutated upstream 1 kb luciferase reporters together into the MCF-7 cells. We found that *HSF2* alone upregulated the luciferase activity of native upstream 1kb 1.9 (± 0.3)-fold, but had no effect on upstream 1 kb with a mutant *HSF2* site. *ZEB1* upregulated the luciferase activity of native upstream 1 kb 6.7 (± 0.7)-fold, but had no effect on *ZEB*1 mutant upstream 1 kb reporter. There was no synergetic effect of these two genes, as co-transfection of the two genes only upregulated the luciferase activity of native upstream 1 kb 2.5 (± 0.2)-fold (Figure 3C).

To further confirm our results, we transfected the *HSF2* and *ZEB*1 overexpression plasmids into MCF-7 cells, and then selected for stable cell lines with G418. Then we compared the expression levels of *miR-96*, *miR-182* and *miR-183* in stable overexpression cell lines with the control cell line, which was transfected with empty vector. Real-time PCR data showed that *miR-96* and *miR-183* were increased 2.7- to 3.8-fold compared to the control cell line, but *miR-182* did not increase very much (Figure 3D). We think the reason why *miR-182* did not increase much is because *miR-182* is far from the transcript regulation area. Although these three miRNAs are transcribed in the same pri-microRNA, the ending of this pri-microRNA is not always the same. Sometimes, it will end before *miR-182* transcription. This result might explain why *miR-182* only increased 4.2 (± 1.1)-fold in tumor samples, but *miR-96* and *miR-183* increased 8.4 (± 1.1)- and 7.5 (± 1.1)-fold in tumor samples. It could also explain why the expression levels of *miR-183* and *miR-182* correlated more strongly sin normal samples, but the correlation dropped dramatically in tumor samples. Because the transcription of *miR-183/-96/-182* was so fast in cancer, some pri-miRNA was not complete.

### Up-regulation of the miR-183/-96/-182 cluster increased cell proliferation and migration and changed the cell cycle profile

**Figure 4:**
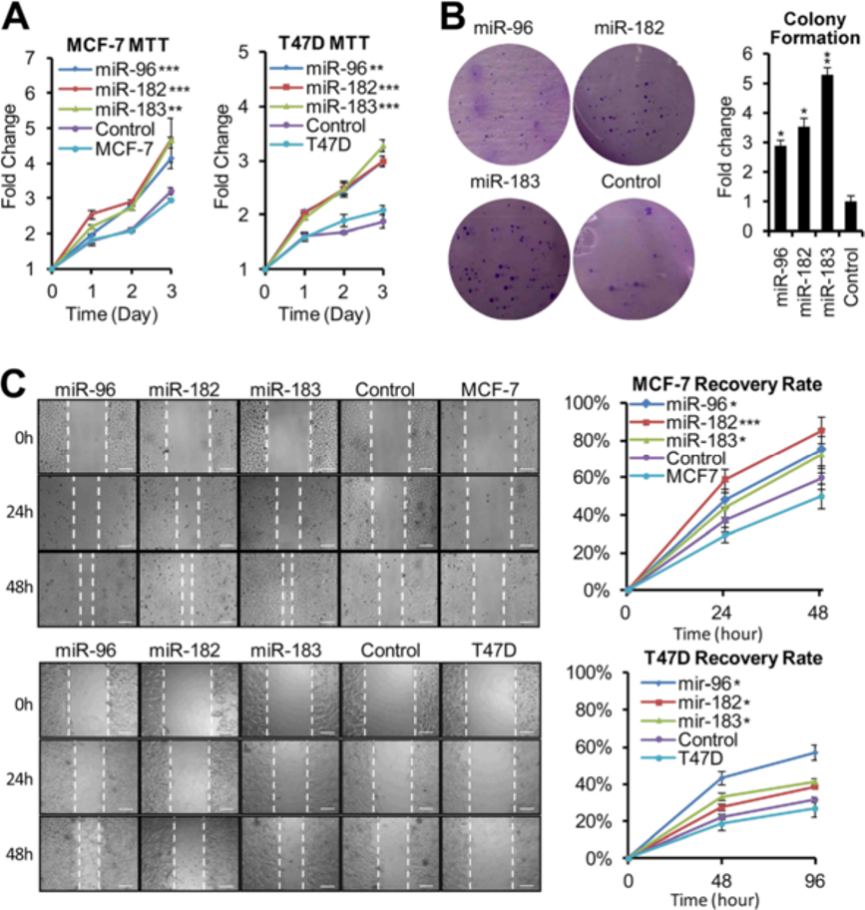
**Up-regulation of the miR-183/-96/-182 cluster increased cell proliferation and migration. (A)** The 3-(4, 5-dimethyl-2-thiazolyl)-2, 5-diphenyl-2H-tetrazolium bromide (MTT) assays showed that *miR-183/-96/-182* cluster overexpression cell lines proliferated more rapidly than the vector control and non-infected cells. Error bars represent SD (n = 4). **(B)** Micrographs (left) and quantification (right) of crystal violet-stained cell colonies in *miR-183/-96/-182* cluster overexpression MCF-7 cell lines and the vector control cells. Error bars represent SD (n = 4). **(C)** Cell wound-healing assays demonstrated that the migration abilities of overexpression cell lines were elevated: left panel, representative micrographs; right panel, quantification graph; upper panel, MCF-7 cells; lower panel, T47D cells. Error bars represent SD (n = 4). Scale bars: 100 μM.

To investigate the biological effects of *miR-183/-96/-182* cluster up-regulation in the development and progression of breast cancer, we generated *miR-96*, *miR-182* and *miR-183* overexpression cell lines in both MCF-7 and T47D cells (Additional file 1: Figure S1). Using MTT assays, we observed that the growth rates of all overexpression cell lines were increased as compared with that of empty vector control or non-transfected cells in both MCF-7 and T47D cells (Figure 4A). Furthermore, in colony formation assays, the increase of colony numbers in MCF-7 overexpression cell lines indicated that ectopically expression of the *miR-183/-96/-182* cluster in MCF-7 cells significantly enhanced anchorage-independent growth (Figure 4B). Furthermore, in both MCF-7 and T47D cells, *in vitro* wound-healing assays demonstrated that the migration abilities of *miR-183, miR-96, and miR-182* overexpression cell lines were elevated, as the non-healed areas were smaller in overexpression cell lines than in control (empty vector) or non-transfected cells (Figure 4C).

**Figure 5:**
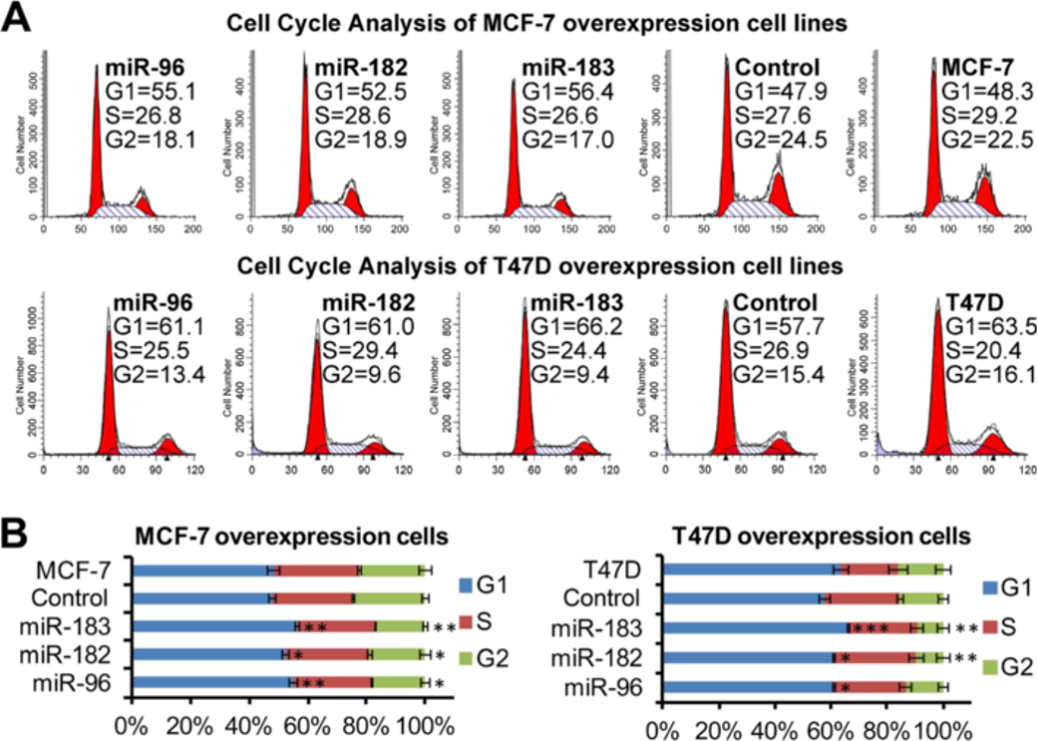
**Up-regulation of miR-183/-96/-182 cluster miRNAs changed the cell cycle profile.** Flow cytometric analysis showed a significant decrease in the percentage of cells in the G2/M peak and an increase in the percentage of cells in the G1/G0 peak of *miR-183/-96/-182* cluster overexpression cell lines compared with the vector control cells and non-infected cells. **(A)** Representative flow cytometric graph of each cell line. **(B)** Quantification graph of the flow cytometric analysis. Error bars represent SD (n = 3).

To further explore the ability of the *miR-183/-96/-182* cluster to promote cell proliferation, we analyzed the cell cycle profile of these overexpression cell lines. In both MCF-7 and T47D cells, flow cytometry results showed a small but significant decrease in the percentage of cells in the G2/M peak and a small but significant increase in the percentage of cells in the G1/G0 peak, the percentage of cells in the S phase was unaltered (Figure 5). These data suggest that the *miR-183/-96/-182* cluster increased the cell proliferation by promoting more rapid completion of mitosis.

### Inhibition of miR-183/-96/-182 cluster miRNAs decreased cell proliferation, and even induced cell death

**Figure 6:**
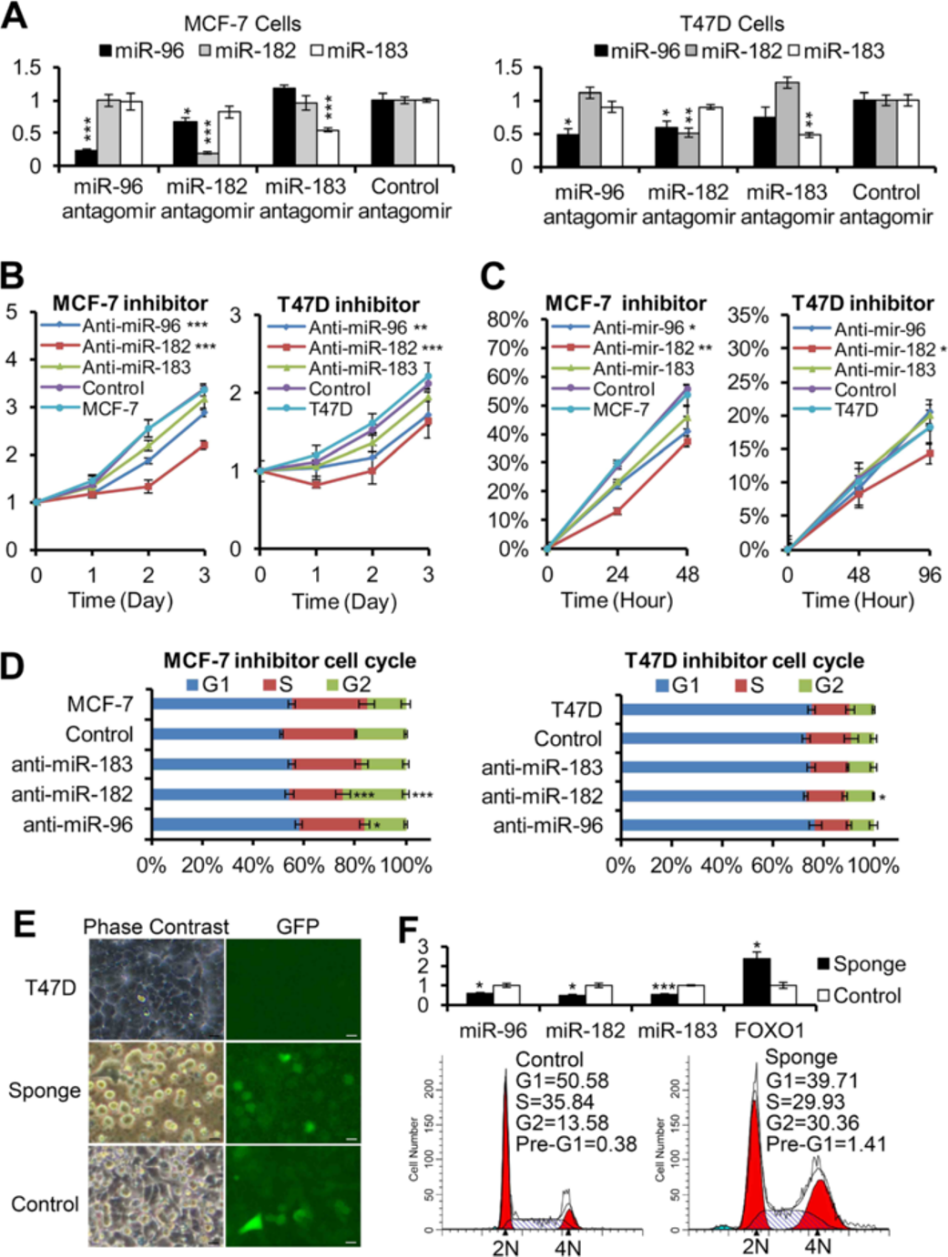
**Inhibition of miR-183/-96/-182 cluster miRNAs decreased cell proliferation, and even induced cell death. (A)** Real-time PCR results showed the knockdown efficiency and specificity of miR-Down™ antagomirs: left panel, MCF-7 cells; right panel, T47D cells. U6 snRNA was used as internal control. Error bars represent SD (n = 4). **(B)** The 3-(4, 5-dimethyl-2-thiazolyl)-2, 5-diphenyl-2H-tetrazolium bromide (MTT) assays showed the cell growth rates of *miR-183/-96/-182* cluster knockdown cells. Error bars represent SD (n = 4). **(C)** Cell wound-healing assays showed the migration abilities of *miR-183/-96/-182* cluster knockdown cells. Error bars represent SD (n = 4). **(D)** Flow cytometric analysis of *miR-183/-96/-182* cluster knockdown cells. Error bars represent SD (n = 3). **(E)** T47D cells infected with *miR-183/-96/-182* cluster sponge lentivirus underwent apoptosis 3 days after transduction: left panels, phase-contrast micrographs of indicated cells; right panels, green fluorescent micrographs of indicated cells. Scale bars: 20 μM. **(F)** Analysis of *miR-183/-96/-182* cluster sponge lentivirus-infected T47D cells: upper panel, inhibition efficiency of *miR-183/-96/-182* cluster sponge lentivirus shown by real-time PCR (error bars represent SD, n = 4; lower panels, flow cytometric graph of indicated cells.

To explore the knockdown effects of *miR-183/-96/-182* cluster miRNAs, we transfected the miR-Down™ antagomirs to the MCF-7 and T47D cells. First, we checked the knockdown efficiency and specificity of these antagomirs. Real-time PCR data showed that each antagomir knocked down its corresponding miRNA efficiently in both MCF-7 and T47D cells. *MiR-182* antagomir also slightly decreased *miR-96* expression, except that there were no cross-reactions. The knockdown efficiency was higher in MCF-7 cells than in T47D cells, and *miR-96* antagomir and *miR-182* antagomir were more efficient than *miR-183* antagomir (Figure 6A). Then, we checked the cell growth rates, cell migrations and cell cycle profiles of these knockdown cells by MTT assay, cell wound-healing assay and cell cycle analysis. MTT assay data showed that knockdown of *miR-96* and *miR-182* decreased the cell growth rates significantly in both MCF-7 and T47D cells. The growth rate of *miR-183* antagomir-treated cells also decreased slightly, but the decrease was not significant (Figure 6B). In MCF-7 cells, the migration abilities of knockdown cells were all decreased although the decrease was not significant for *miR-183* antagomir-treated cells. However, in T47D cells, only the *miR-182* antagomir led to the decrease of migration; the migration ability of *miR-96* and *miR-183* antagomir-treated cells remained the same (Figure 6C). Furthermore, cell cycle analysis demonstrated a significant increase in G2/M phase and a decrease in S phase for cells treated with *miR-182* antagomir in MCF-7 cells. Knockdown of *miR-96* also decreased the percentage of cells in S phase slightly in MCF-7 cells, but in T47D cells the cell cycle profiles were not changed except for a slight increase in G2/M phase after *miR-182* antagomir treatment (Figure 6D). We think the different behavior of MCF-7 and T47D cells after antagomir treatment was related to the knockdown efficiency. As the knockdown efficiency was higher in MCF-7 cells, the growth rate and migration ability of MCF-7 cells were seriously inhibited by the antagomir. Knockdown of *miR-183* did not affect the cell profiles too much either in MCF-7 or in T47D cells. This phenomenon could be explained by the inefficient knockdown and the compensatory effect. *MiR-96* and *miR-182* might have substituted partial function of *miR-183* and compensated the loss of *miR-183*.

To further examine the biological effect of the *miR-183/-96/-18*2 cluster as a whole on breast cancer cells, we generated *miR-183/-96/-182* cluster sponge lentivirus (Additional file 2: Figure S2), and infected T47D cells with this vector. First, we checked the inhibition efficiency of this virus by real-time PCR. Compared to the empty vector, the expressions of *miR-183/-96/-182* cluster miRNAs were dropped to a half after sponge lentivirus transduction; and the expression of *FOXO1*, which was a generally acknowledged target gene of the *miR-183/-96/-182* cluster, was increased about 2-fold after sponge lentivirus transduction (Figure 6F upper panel). We found that T47D cells underwent cell death and apoptosis after transduction. Three days after transduction, the cells became round and detached (Figure 6E). Cell cycle analysis showed an increase in the percentage of cells in the G2/M peak and pre-G1 peak and a decrease in the percentage of cells in the G1/G0 peak, indicating that inhibition of *miR-183/-96/-182* induced G2/M arrest and apoptosis (Figure 6F lower panel).

As MCF-7 and T47D cells are both luminal breast cancer, we also tested the miRNA knockdown effects in basal-like breast cancer cells, such as BT-20 (Basal A) and MDA-MB-231 (Basal B) cells. We found basal-like cells were more sensitive to the depletion of the *miR-183/-96/-182* cluster than the luminal-like cells. MTT experiments showed BT-20 ceased proliferation and underwent cell death after knockdown of *miR-96*, *miR-182* or *miR-183* (Additional file 7: Figure S4A, B). MDA-MB-231 cells underwent cell death and apoptosis after transduction of *miR-183/-96/-182* cluster sponge lentivirus. The cells became round and detached 3 days after transduction, and the cell cycle analysis showed that pre-G1 cells, which represented the apoptotic cells, were increased in knockdown cells (Additional file 7: Figure S4C, D).

### MiR-183 targeted the RAB21 gene directly in breast cancer

**Figure 7:**
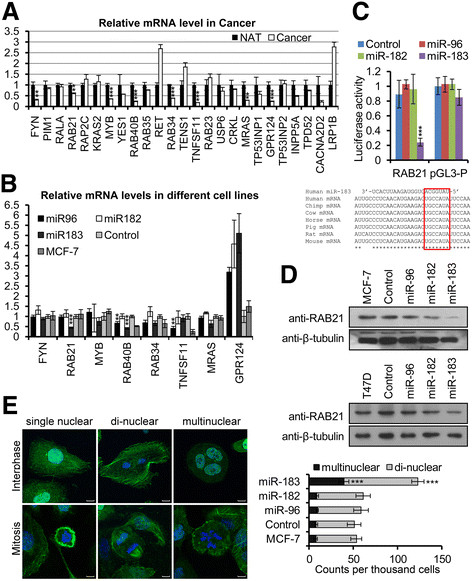
**Identification of candidate targets of miR-183/-96/-182 cluster miRNAs.** Predicted miR-183/-96/-182 targets are listed in Additional file 4: Table S2 where their NCBI reference sequence, putative binding miRs and detection primers are also provided. **(A)** Comparison of candidate target mRNA levels between breast cancer samples and their normal adjacent tissue (NAT) by real-time PCR; glyceraldehyde-3-phosphate dehydrogenase (GAPDH) was used as an internal control. Error bars represent SD (n = 3). **(B)** Confirmation of target genes by real-time PCR in *miR-183/-96/-182* cluster overexpression stable MCF-7 cell lines and control cells; GAPDH was used as an internal control. Error bars represent SD (n = 3). **(C)** Confirmation of miR-183/-96/-182 cluster targets by luciferase assay. All data were normalized to those obtained with the pGL3-Promoter vector alone. Error bars represent SD (n = 3): lower panel, sequences of miR-183 and its target sequences in the 3'-UTR of different species. **(D)** Protein levels of *RAB21* in stable cell lines were documented by western blot with an anti-*RAB21* antibody. β-Tubulin was used as the internal control; upper panel, MCF-7 cells; lower panel, T47D cells. **(E)** Phalloidin and 4',6-diamidino-2-phenylindole (DAPI) counterstaining results showed that the bi- and multinuclear cells were accumulated in *miR-183* over-expressed MCF-7 cells; left panels, representative micrographs of single, bi- and multinuclear cells in both interphase and mitosis; right panel, quantification of bi- and multinuclear cells in different cell lines. Error bars represent SD (n = 5). Scale bars: 10 μM.

To better understand the biological roles of the *miR-183/-96/-182* cluster miRNAs in breast cancer, we compiled a list of putative target genes of the *miR-183/-96/-182* cluster that were dysregulated in breast cancer by using three computational target prediction-algorithms: PicTar, TargetScan 5.1 and MicroCosm (Additional file 4: Table S2). As mammalian miRNAs regulate target genes predominantly by acting to decrease target mRNA levels [24], we first compared the mRNA levels of those genes between breast cancer sample and its NAT by real-time PCR with *GAPDH* used as an internal control. Eight genes out of twenty-five candidates showed significantly decreased expression in breast cancer (Figure 7A). To validate the eight candidates, we checked their mRNA levels in the *miR-96*, *miR-182* and *miR-183* MCF-7 overexpression cell lines. Compared to empty vector control cells, *RAB21* was decreased in the *miR-183* overexpression cell line; *RAB40B* was decreased in *miR-96* and *miR-183* overexpression cell lines and *TNFSF11* was decreased in the *miR-96* overexpression cell line (Figure 7B).

Because *RAB21* was the predicted target gene of *miR-183*, we focused our efforts on this target. *RAB21*, which belongs to the *Rab* family of monomeric GTPases, plays a role in integrin internalization and recycling. As a result, the encoded protein is involved in cytokinesis during the mitosis. Loss of *RAB21* in the tumor induces chromosome number aberrations and malignancy [25]. To further evaluate the role of *miR-183* in regulating *RAB21*, we generated luciferase *reporters with 33 bp of the predicted target regions from the 3'-UTR of RAB21, and co-*transfected the reporter with *miR-96*, *miR-182* and *miR-183* overexpression vectors and empty vector. The luciferase assay results showed that *miR-183* repressed luciferase activity dramatically in the reporter derived from the *RAB21-*targeted region compared with the empty vector, and *miR-96* and *miR-182* had no effect on the luciferase activity of the *RAB21* reporter. As a negative control, the luciferase activity of cells containing the empty pGL3-Promoter vector was not affected by any *miR-183/-96/-182* cluster miRNA (Figure 7C). The protein levels of *RAB21* were also determined in both MCF-7 and T47D overexpression cell lines with β-tubulin used as an internal control. The data showed that *RAB21* protein was significantly decreased in the *miR-183* overexpression cell lines, but not in *miR-96* and *miR-182* overexpression cell lines compared with the empty vector control cell lines and wild-type cells (Figure 7D). As loss of *RAB21* in the tumor would induce chromosome number aberrations, we checked the nucleus aberration in *miR-183/-96/-182* cluster overexpression MCF-7 cells. Phalloidin and DAPI counterstaining results showed that the bi- and multinuclear cells were accumulated in *miR-183* overexpressed cells but not in *miR-96* and *miR-182* overexpressed cells (Figure 7E). All these data indicated that *miR-183* targeted the *RAB21* gene directly in breast cancer and induced aneuploidy.

## Discussion

The *MiR-183/-96/-182* cluster is a conserved polycistronic miRNA cluster that is highly expressed in several tumor types. Although it is well known that the expression level of this miRNA cluster is increased in breast cancer, its biological roles and the regulatory mechanisms governing *MiR-183/-96/-182* expression in breast cancer are still unclear. Here, we report that *miR-96*, *miR-182* and *miR-183* expression levels are significantly higher in breast cancer compared to the NAT, and the transcription pattern of *miR-183/-96/-182* is irregular in breast cancer as the correlation between *miR-182* and *miR-183* expression dropped dramatically in tumor samples. The expression of *miR-183/-96/-182* is not upregulated in a specific breast cancer subtype. It is overexpressed in all kinds of breast cancer - ductal or lobular, luminal or basal, early-stage or late-stage - but there are some differences in their expression patterns. For example, *miR-96* and *miR-183* were lower in lobular carcinoma than in ductal carcinoma and other types of carcinoma. The levels of *miR-96* and *miR-183* were also lower in ER+ and PR+ cancers than in ER− and PR− cancers, but *miR-182* was almost the same, even a little higher in ER+ cancers. Among the four different subtypes of breast cancer, *miR-96* and *miR-183* levels were higher in HER2-enriched breast cancers than other types; *miR-182* was lower but *miR-183* was higher in basal-like breast cancers than other types of breast cancer. We also compared the miRNA expression levels in different breast cancer cell lines based on their molecular markers. We found that *miR-96* is only upregulated in SK-BR-3 and BT-20 cells, whereas *miR-182* and *miR-183* are upregulated in most of the breast cancer cell lines tested except for MDA-MB-231. Basically, the cell line data closely match the clinical analysis. *MiR-96* and *miR-183* levels are higher in HER2-enriched cell line SK-BR-3. *MiR-96* is lower in ER+ and PR+ cancers than in ER- and PR- cancers. *MiR-182* is higher in luminal breast cancer than basal breast cancer. MDA-MB-231 is the only exception. It is an ER- and PR- cancers, but its expression of *miR-183/-96/-182* is low. Because MDA-MB-231 is a basal B/claudin-low breast cancer cell line, which lacks common epithelial cell features and most closely resembles the mammary epithelial stem cell [26], we think its regulation of *miR-183/-96/-182* is different to other breast cancer cell lines. Our data were similar to those reported by Riaz and colleagues. Based on their work, 51 human breast cancer cell lines were divided into two groups: the first major group included 33 cell lines, which was a luminal-like group; the second minor group included 18 cell lines, which was a basal-like group. Seventeen miRNAs, which included *miR-182,* showed significantly higher expression in the major cluster compared with the other miRNAs. They also found that the expression of *miR-183/-96/-182* is low in MDA-MB-231 cells [23]. Although the *miR-183/-96/-182* cluster is transcribed in the same pri-miRNA, the expression profile of each miRNA varies between different cell lines, which indicate that their subsequent processing or stability are regulated in different ways. An interesting phenomenon is that from the 102 patient samples of TCGA dataset, *miR-182* only increases 4.2 (± 1.1)-fold in tumor samples, but *miR-96* and *miR-183* increase 8.4 (± 1.1)- and 7.5 (± 1.1)-fold in tumor samples. The correlation between the expressions of *miR-182* and *miR-183* dropped dramatically in tumor samples. This phenomenon was also confirmed in HSF2 and ZEB1 overexpression cell lines, as the expressions of *miR-96* and *miR-183* were increased significantly, but not *miR-182*. We think it is because the transcription of *miR-183/-96/-182* is very fast in cancer; some pri-miRNA is not complete and the transcription stalls before *miR-182*.

We also identified two transcriptional factors that regulate the transcription of the *miR-183/-96/-182* cluster, *ZEB1* and *HSF2. ZEB1*, which is a zinc finger transcription factor, is involved in the epithelial-mesenchymal transition and promotes metastasis in cancer [27],[28]. Although most work has concentrated on the capacity of *ZEB1* to repress gene expression, several groups demonstrated that *ZEB1* can also activate transcription of downstream targets [28],[29]. *HSF2* binds heat shock promoter elements (HSE) and activates transcription. Although there is little evidence on the involvement of *HSF2* in tumorigenesis, it can play a role indirectly by modulating *HSF1* [30]. Previous studies also report that *HSF2* regulates the proto-oncogene c-fos and may be involved in tumorigenesis [31]. Our findings show that *ZEB1* and *HSF2* activate the transcription of the *miR-183/-96/-182* cluster, which gives us new insights into how *ZEB1* and *HSF2* enhance tumorigenesis.

The biological role of the *miR-183/-96/-182* cluster in breast cancer is complicated. In our experience, this cluster functions more like an oncogene in breast cancer as it increases cancer cell proliferation and migration. Most previous and recent publications support this conclusion, especially for *miR-182*, which has been confirmed by many groups to induce breast cancer metastasis [6],[32]-[34]. *Mir-96* is also proposed to be an onco-miRNA in breast cancer [5],[6], but the role of *miR-183* is more complex. It represses the expression of *EGR1* and functions as an oncogene in breast cancer [35], but it also targets the *Ezrin* gene and inhibits cell migration in T47D cells [12]. Our results support a pro-oncogenic role for *miR-183* in breast cancer, because upregulated expression of *miR-183* by lentivirus in MCF-7 cells induces cell proliferation and migration. The effects of knockdown of *miR-183/-96/-182* cluster are more complicated, and depend on the knockdown efficiency and specificity. We did not observe obvious changes after inhibition of *miR-183,* but we found a significant decrease in cell growth rates and S phase cell percentages in *miR-96* and *miR-182-*inhibited cells. Two reasons can explain these results. First, the knockdown efficiency of *miR-183* antagomir is lower than *miR-96* and *miR-182* antagomir. Second, *miR-96* and *miR-182* target *FOXO1*, but *miR-183* does not [6]. *MiR-96* and *miR-182* might compensate partial functions of *miR-183*, but *miR-183* cannot replace the function of *miR-96* and *miR-182* on inhibition of *FOXO1*.

Long-term inhibition of three miRNAs by sponge elements induced cell death and apoptosis in T47D cells, but we did not detect apoptosis with a single antagomir transfection. Inhibition of two or three of the cluster members at one time induced apoptosis, though some of them were not statistically significant (Additional file 8: Figure S5). These data indicate that these three miRNAs are redundant; they may be complimentary to each other. Knockdown of *miR-183* had little effect on its own, but it had collaborative effects with the other two miRNAs.

We identified *RAB21* as a target gene of *miR-183* in both mRNA and protein levels, and also confirmed that overexpression of *miR-183* induced accumulation of bi- and multinuclear cells. *RAB21* is involved in the targeted trafficking of integrins via its association with integrin alpha tails. As a consequence, *RAB21* regulates cell adhesion and migration [36]. In mitotic cells, integrin trafficking regulated by *RAB21* is necessary for cytokinesis and cytokinesis failure will induce aneuploidy and oncogenic transformation [25],[37]. This information may answer the question why *miR-183* has dual effects in breast cancer. In some cases, repression of *RAB21* results in decreased cell mobility, but in other cases, repression of *RAB21* may lead to cytokinesis failure and aneuploidy. The 3'-UTR of *RAB21* matches the seed sequence of *miR-183*, but not *miR-96* nor *miR-182*. So, only *miR-183* can inhibit the expression of *RAB21*. As the phenotype is similar no matter which of the three miRNAs is overexpressed in MCF-7 and T47D cells, *RAB21* down regulation itself is not enough to explain the phenotype. Some other mechanisms are also involved in the regulation of cell proliferation and migration. For example, inhibition of *FOXO1* by *miR-96* and *miR-182* will increase cell proliferation.

We identified two regulators (*ZEB1* and *HSF2*) and one target gene (*RAB21*) for the *miR-183/-96/-182* cluster in breast cancer cell lines. How do they work in clinical samples? We looked for correlation between *miR-183/-96/-182* cluster miRNAs and their target/regulators by analysis of 508 clinical samples from TCGA data (Additional file 9). Because the correlations between miRNAs and their targets/regulators are not simply negative or positive correlations, we did not find any direct correlations between these miRNAs and the expressions of *HSF2*, *ZEB1* and *RAB21* based on the TCGA data analysis. But there were some interesting correlations between them in different subtypes. *MiR-96* and *miR-183* weree lower in ER+ and PR+ breast cancers than ER- and PR- breast cancers; in the meantime, their regulator, *HSF2* level was lower and their target, *RAB21* level, was higher in ER+ and PR+ breast cancers than ER- and PR- breast cancers (Additional file 4: Table S4). Subtype analysis also confirmed our findings. *HSF2* level was high in basal breast cancers, which are *miR-183-*enriched breast cancers; and *RAB21* level was low in HER2 and basal breast cancers, which are *miR-96-* and/or *miR-183-*enriched breast cancers (Additional file 4: Table S5). *MiR-182* was not strongly correlated with the levels of *HSF2* because its transcription is not controlled by *HSF2* (Figure 3D)*.* There is still a complicated phenomenon that requires explanation, which is that the *ZEB1* level was negatively correlated with *miR-96* and *miR-183* (Additional file 4: Table S4, S5)*.* In MCF-7 cells, *ZEB1* upregulates the expressions of *miR-96* and *miR-183* (Figure 3)*,* and Graham *et al*. also report that *ZEB1* is more expressed in ER/PR- breast cell lines than ER/PR+ breast cell lines [38]. However, in clinical samples, *ZEB1* was enriched in ER/PR+ samples. Considering *ZEB1* is a transcription factor that can either activate or repress its target genes, we think it functions differently in breast cancer cell lines and breast cancer patients. In patients, *ZEB1* may repress the transcription of *miR-183/-96/-182* cluster. This conclusion needs further work for confirmation, but nevertheless, *ZEB1* plays an important role in the regulation of *miR-183/-96/-182* cluster.

## Conclusion

We found that the *miR-183/-96/-182* cluster is highly expressed in most breast cancers, and its transcription is disordered in breast cancers. The *miR-183/-96/-182* cluster is transcribed in the same pri-miRNA and its transcription is regulated by *ZEB1* and *HSF2*. It increases breast cancer cell proliferation, promotes cell migration and is essential for cell survival. Also, *miR-183* targets the *RAB21* gene directly in breast cancer. In summary, the *miR-183/-96/-182* cluster is upregulated in most breast cancers. It functions as an oncogene in breast cancer as it increases cell proliferation and migration. This can be partially explained by the inhibition of tumor suppressor gene *RAB21.*

The bioinformatics tools used in this manuscript were the miRBase Sequence Database [39]; the TCGA dataset [40]; the ENCODE Project [41]; TFSEARCH [42]; PicTar [43]; TargetScan 5.1 [44], and MicroCosm [45].

## Authors' information

QZ is a geneticist and molecular biologist whose research interests focus on cancer and stem cells. Dr QZ received his BSc degree from Wuhan University, China (2003) and his PhD from Texas A&M University, Houston, USA (2009) where he studied signal pathways in cancer and stem cells. Dr QZ performed his postdoctoral training at the department of pharmacology in Case Western Reserve University where he started to work on microRNA. He became Associate Professor in 2011 at the School of Pharmaceutical Science in Central South University, China, and got the foundation from National Natural Science Foundation of China in 2012. QZ is a member of the Chinese Pharmacological Society and his recent work focuses on the roles of microRNA in cancer.

## Additional files

## Electronic supplementary material

Additional file 1: Figure S1.: Creation of miR-183/96/182 stable cell lines. (PDF 7 MB)

Additional file 2: Figure S2.: Creation of miR-183/96/182 cluster sponge lentivirus. (PDF 2 MB)

Additional file 3: Figure S3.: Test of the specificity of cluster probes by LNA-based Northern Blot. (PDF 611 KB)

Additional file 4: **Supplementary tables. Table S1.** miRNA mimics used in LNA-based northern blot. **Table S2.** Primer sets for predicted miR-183/-96/-182 cluster target genes. **Table S3.** Primer sets for pri-miRNA transcription screening. **Table S4.** The correlations between miRNAs' targets/regulators and surface markers. **Table S5.** miRNAs' targets/regulators in different molecular subtypes of breast cancer. (DOC 84 KB)

Additional file 5: MiRNAs in breast cancers and their matched normal controls. (XLS 60 KB)

Additional file 6: Clinical features and miRNAs. (XLS 181 KB)

Additional file 7: Figure S4.: Test of the miRNA knockdown effects in basal-like breast cancer cells. (PDF 4 MB)

Additional file 8: Figure S5.: Inhibition two or three of the cluster members at one time induced apoptosis in T47D cells. (PDF 1 MB)

Additional file 9: Targets and regulators. (XLS 138 KB)

Below are the links to the authors’ original submitted files for images.

Authors’ original file for figure 1

Authors’ original file for figure 2

Authors’ original file for figure 3

Authors’ original file for figure 4

Authors’ original file for figure 5

Authors’ original file for figure 6

Authors’ original file for figure 7

## Abbreviations

BAC: bacterial artificial chromosome
bp: base pairs
ChIP: chromatin immunoprecipitation
CT: cycle threshold
DAPI: 4',6-diamidino-2-phenylindole
DMEM: Dulbecco's modified Eagle's medium
DMSO: dimethyl sulphoxide
ELISA: enzyme-linked immunosorbent assay
ER: estrogen receptor
FBS: fetal bovine serum
FOXO: forkhead box O
GFP: green fluorescent protein
HER2: human epidermal growth factor receptor-2
HSE: heat shock promoter elements
miRNA: microRNA
MOI: multiplicity of infection
MTT: 3-(4, 5-dimethyl-2-thiazolyl)-2, 5-diphenyl-2H-tetrazolium bromide
NAT: normal adjacent tissues
NBEC: normal breast epithelial cells
PBS: phosphate-buffered saline
PI: propidium iodide
PR: progesterone receptor
RT: reverse transcription
TSS: transcription start site
UTR: untranslated region
