# Supplementary material for: MiR-183/-96/-182 cluster is up-regulated in most breast cancers and increases cell proliferation and migration
Source: Breast Cancer Res. 2014 Nov 14;16:473. doi: 10.1186/s13058-014-0473-z (PMC4303194; doi:10.1186/s13058-014-0473-z)
Supplement: Supplementary file 1 — Additional file 1: Figure S1.: Creation of miR-183/96/182 stable cell lines. (PDF 7 MB) [file 13058_2014_473_MOESM1_ESM.pdf]

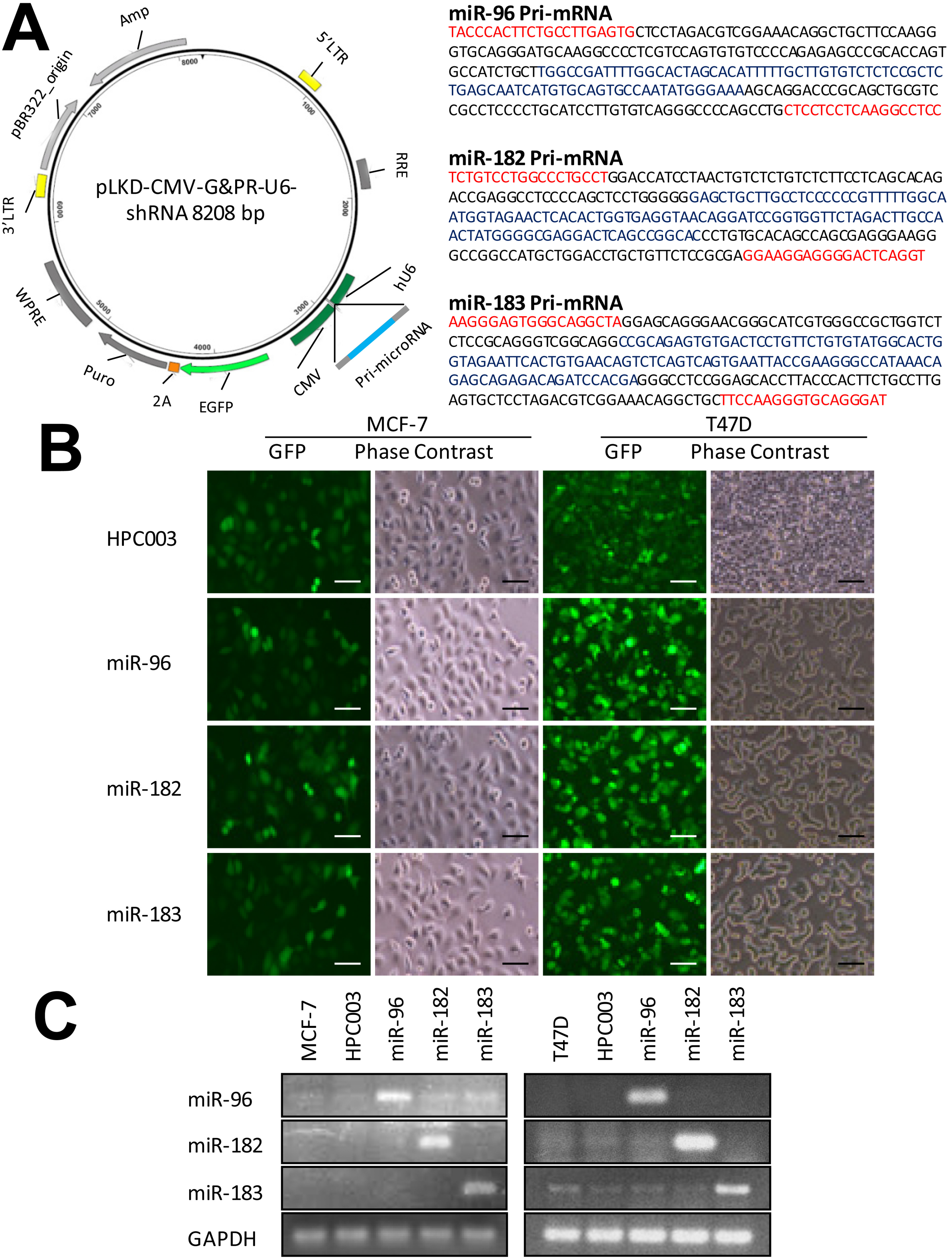

Figure S1 Creation of miR-183/96/182 stable cell lines. (A) Consctruction of the *miR-183/96/182* overexpression lentivirus: Left panel shows the schematic representation of the pLKD-CMV-G&PR-U6-shRNA lentivirus; right panel shows the inserted partial pri-miRNA sequences of miR-183/96/182 cluster miRNAs. Letters in blue indicate the pre-miRNA sequences; letters in red denote cloning and detecting primer sequences. **(B)** GFP fluorescent and phase contrast images of the stable cell lines illustrated the efficiency of lentivirus infection. Scale bars: 50 μm **(C)** Confirmation of the pri-miRNA expression levels in stable cell lines by RT-PCR detection. GAPDH was used as internal control.
