# Supplementary material for: MiR-183/-96/-182 cluster is up-regulated in most breast cancers and increases cell proliferation and migration
Source: Breast Cancer Res. 2014 Nov 14;16:473. doi: 10.1186/s13058-014-0473-z (PMC4303194; doi:10.1186/s13058-014-0473-z)
Supplement: Supplementary file 2 — Additional file 2: Figure S2.: Creation of miR-183/96/182 cluster sponge lentivirus. (PDF 2 MB) [file 13058_2014_473_MOESM2_ESM.pdf]

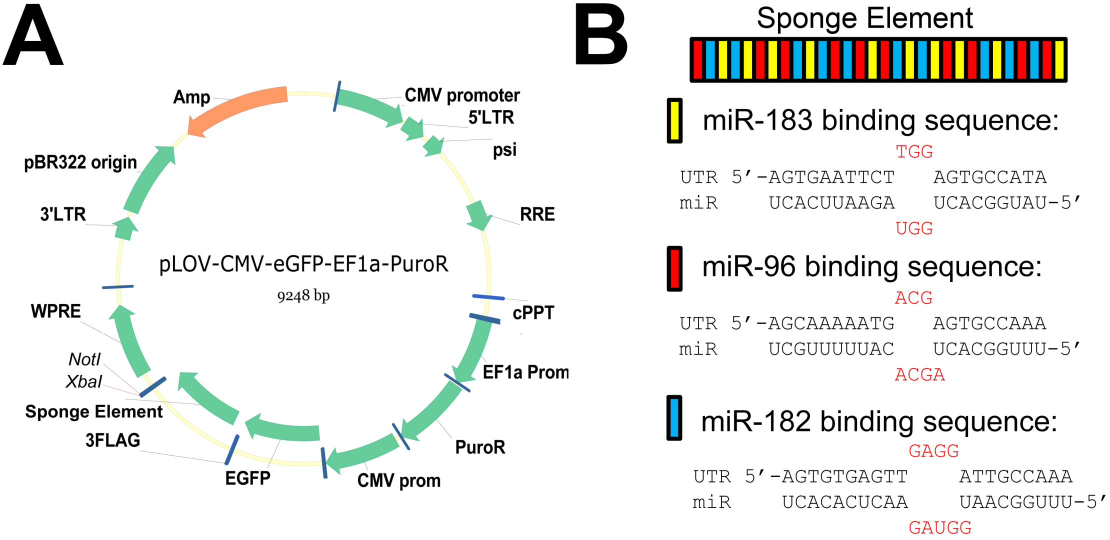

Figure S2 Creation of miR-183/96/182 cluster sponge lentivirus. (A) Schematic representation of the pLOV-CMV-eGFP-EF1a-PuroR lentivirus. **(B)** Upper panel shows the schematic representation of the sponge element: 10 copies of binding sites for each miR-183/96/182 cluster miRNA were inserted into the 3'-UTR of EGFP. The construct was under control of CMV promoter. Binding sequences of miR-183, miR-96 and miR-182 are shown in the lower panel.
