# Supplementary figures and images for: MiR-183/-96/-182 cluster is up-regulated in most breast cancers and increases cell proliferation and migration
Source: Breast Cancer Res. 2014 Nov 14;16:473. doi: 10.1186/s13058-014-0473-z (PMC4303194; doi:10.1186/s13058-014-0473-z)

# Antisense DNA

Probes

miR-96

miR-182  
(human)

miR-183

miR-182  
(mouse)

miR-96

miR-182  
(human)

miR-183

miR-182  
(mouse)

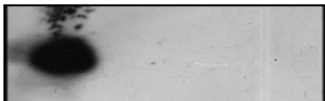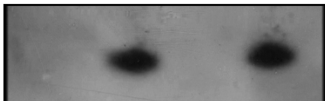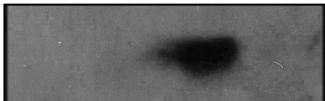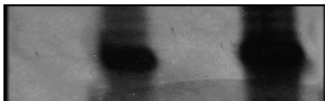

Supplement: Supplementary file 3 — Additional file 3: Figure S3.: Test of the specificity of cluster probes by LNA-based Northern Blot. (PDF 611 KB) [file 13058_2014_473_MOESM3_ESM.pdf]

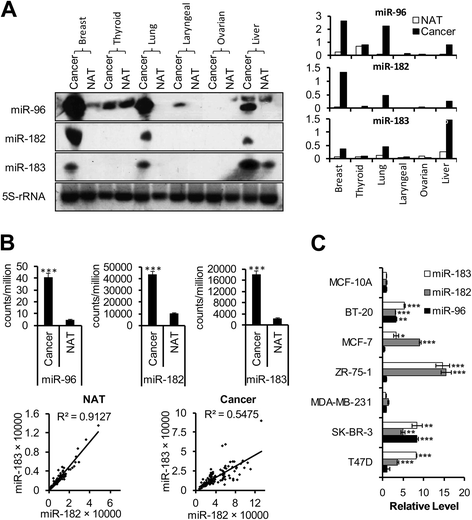

Supplement: Supplementary file 10 — Authors’ original file for figure 1 [file 13058_2014_473_MOESM10_ESM.gif]

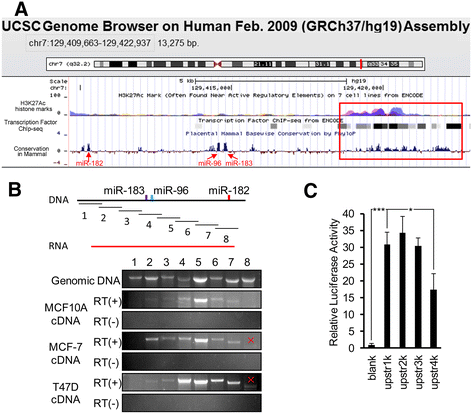

Supplement: Supplementary file 11 — Authors’ original file for figure 2 [file 13058_2014_473_MOESM11_ESM.gif]

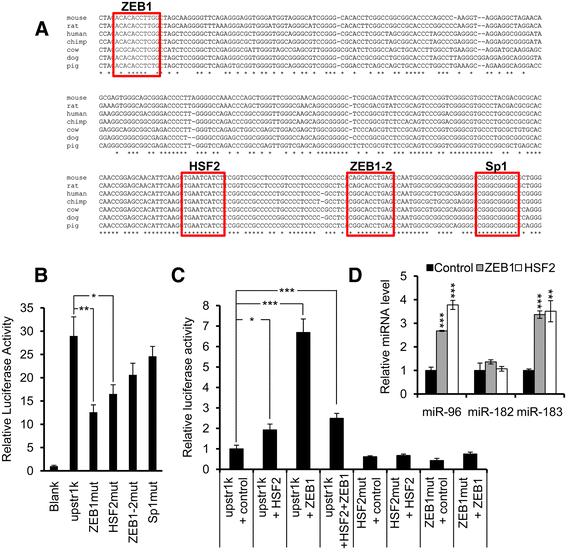

Supplement: Supplementary file 12 — Authors’ original file for figure 3 [file 13058_2014_473_MOESM12_ESM.gif]

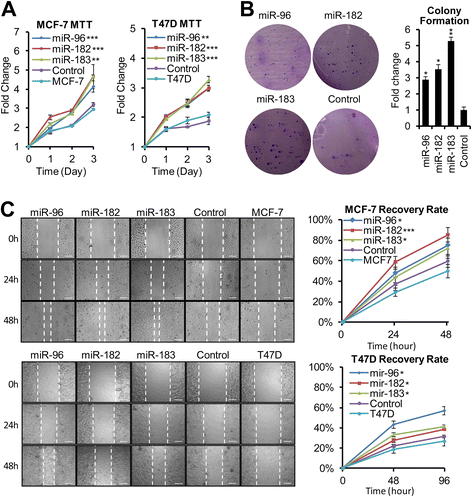

Supplement: Supplementary file 13 — Authors’ original file for figure 4 [file 13058_2014_473_MOESM13_ESM.gif]

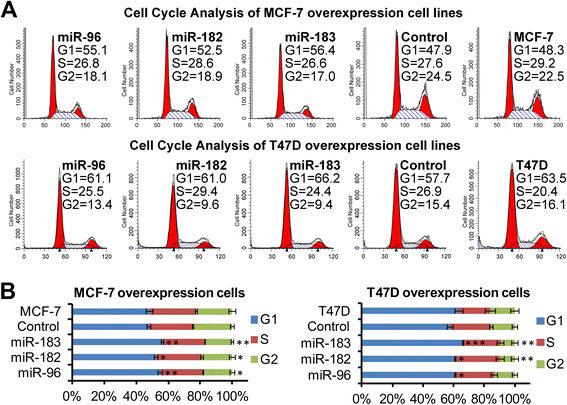

Supplement: Supplementary file 14 — Authors’ original file for figure 5 [file 13058_2014_473_MOESM14_ESM.gif]

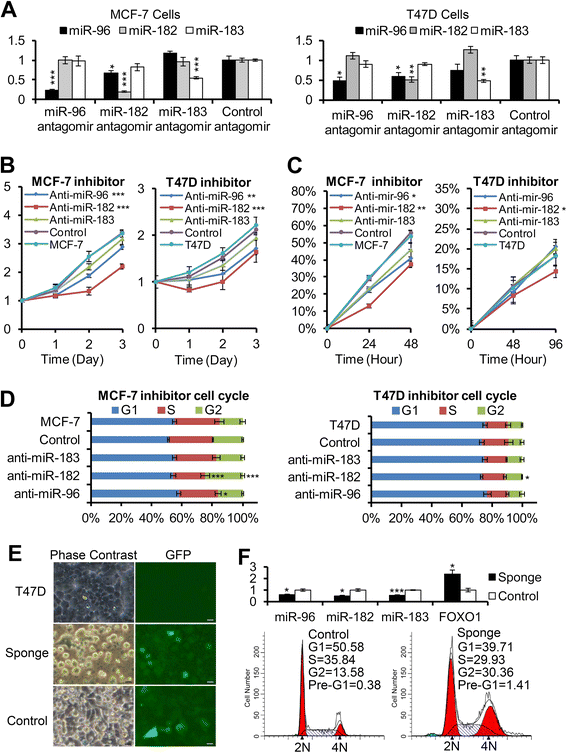

Supplement: Supplementary file 15 — Authors’ original file for figure 6 [file 13058_2014_473_MOESM15_ESM.gif]

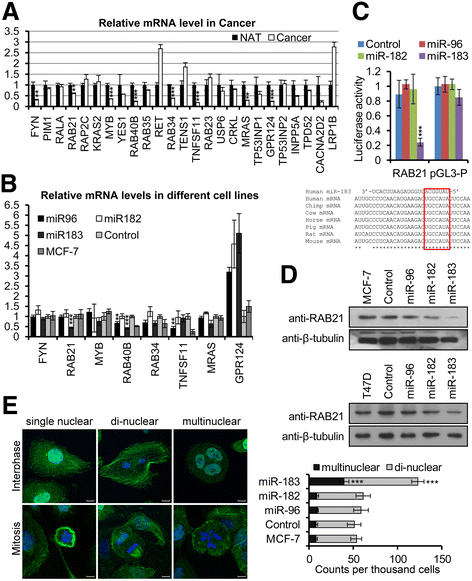

Supplement: Supplementary file 16 — Authors’ original file for figure 7 [file 13058_2014_473_MOESM16_ESM.gif]
