# Supplementary material for: MiR-183/-96/-182 cluster is up-regulated in most breast cancers and increases cell proliferation and migration
Source: Breast Cancer Res. 2014 Nov 14;16:473. doi: 10.1186/s13058-014-0473-z (PMC4303194; doi:10.1186/s13058-014-0473-z)
Supplement: Supplementary file 4 — Additional file 4: Supplementary tables. Table S1. miRNA mimics used in LNA-based northern blot. Table S2. Primer sets for predicted miR-183/-96/-182 cluster target genes. Table S3. Primer sets for pri-miRNA transcription screening. Table S4. The correlations between miRNAs' targets/regulators and surface markers. Table S5. miRNAs' targets/regulators in different molecular subtypes of breast cancer. (DOC 84 KB) [file 13058_2014_473_MOESM4_ESM.doc]

**SUPPLEMENTARY TABLE**

**Table S1. miRNA mimics used in LNA-based Northern Blot.**1

| **Name** | **Sequence** |
| --- | --- |
| Mimic-mmu-miR-182 | TTTGGCAATGGTAGAACTCACACCG |
| Mimic-hsa-miR-96 | TTTGGCACTAGCACATTTTTGCT |
| Mimic-hsa-miR-182 | TTTGGCAATGGTAGAACTCACACT |
| Mimic-hsa-miR-183 | TATGGCACTGGTAGAATTCACT |

^1^Mimic oligonucleotides simulating the endogenous miRNA sequences were used as positive controls.

**Table S2. Primer sets for predicted miR-183/-96/-182 cluster target genes^1^**

| **Name** | **mRNA** | **miRNA** | **Primer sets** |
| --- | --- | --- | --- |
| FYN | NM_002037 | miR-96 | Forward: CTGCGATCAGCAAACATTCT  Reverse: GGGAACTTTGCACCTTGTCT |
| PIM1 | NM_002648 | miR-183 | Forward: GGGTCTCTTCAGAATGTCAGC  Reverse: TGGATCTCAGCAGTTTCCTG |
| RALA | NM_005402 | miR-183 | Forward: AATAAGCCCAAGGGTCAGAA  Reverse: AGTCCTCCACAAACTCATCGT |
| RAB21 | NM_014999 | miR-183 | Forward: CACTCTGCAGGCATCATTCT  Reverse: TCGCTCCATTTGAATCTCTG |
| RAP2C | NM_021183 | miR-183 | Forward: ACTGACCAGTTTGCCTCCAT  Reverse: TCATTGGCTTGATATCCTGAAA |
| KRAS | NM_004985 | miR-96, miR-182 | Forward: GGAGAGAGGCCTGCTGAA  Reverse: TTGGATCATATTGGTCCACAA |
| MYB | NM_005375 | miR-96, miR-182 | Forward: AGCAGGTGCTACCAACACAG  Reverse: CTCCCAAACAGGAAACAGGT |
| YES1 | NM_005433 | miR-96, miR-182, miR-183 | Forward: GCTCCTGAAGCTGCACTGTA  Reverse: ACGGTTCACCATACCTGGAT |
| RAB40B | NM_006822 | miR-96, miR-182 | Forward: TGTGCAATTTCAACATCACAG  Reverse: CAAGCTCAGCACCTTGCTC |
| RAB35 | NM_006861 | miR-96, miR-182 | Forward: GGGACTACGACCACCTCTTC  Reverse: GGTGATGTAGCTGCCTGAGA |
| RET | NM_020630 | miR-96, miR-182 | Forward: CCTATCCTGGGATTCCTCCT  Reverse: ATCAGGCGGTACATCTCCTC |
| RAB34 | NM_031934 | miR-96, miR-182 | Forward: AGAGGAGCTCAAGCCATCAT  Reverse: GAGAAGCACACTGGAAGGGT |
| TENS1 | NM_022748 | miR-96, miR-182 | Forward: GCAGCTGAACAAGAAAGCTG  Reverse: GCTCCCGAAATATGGTTCAT |
| TNFSF11 | NM_003701 | miR-182 | Forward: AGAGCAGAGAAAGCGATGGT  Reverse: TGTCGGTGGCATTAATAGTGA |
| RAB23 | NM_016277 | miR-96, miR-182 | Forward: TTTGGAGCGACAAATTCAAG  Reverse: CCTGGGCTCCTCGATAGTAG |
| USP6 | NM_004505 | miR-96, miR-182 | Forward: AGCATGTGGTACCCAAGTCA  Reverse: GAGATCCCGTCAATCAGGTT |
| CRKL | NM_005207 | miR-96, miR-182 | Forward: CTTTGCCATCCACACAGAAT  Reverse: TCACCAACCTCTAATGCCAA |
| MRAS | NM_012219 | miR-96, miR-182 | Forward: CGTCAAAGACAGGGAGTCATT  Reverse: TATTGTGTTTGGTCGCCATT |
| TP53INP1 | NM_033285 | miR-96, miR-182 | Forward: CGCAGCTACCTCAGCAGTC  Reverse: AGGTGAAAAGCAAGAAGAGTCA |
| GPR124 | NM_032777 | miR-96, miR-182 | Forward: TTCCACATCAAGAACAGCGT  Reverse: ATTTCGGAAGACGAGCAGTT |
| TP53INP2 | NM_021202 | miR-96, miR-182 | Forward: CTCCCAGCTGTTTGGATCAC  Reverse: GCTGAAGAAGAGGCTGGAGA |
| INPP5A | NM_005539 | miR-96, miR-182 | Forward: TCACCTATGACCACATTGGG  Reverse: TCACTGCACGACACAACACT |
| TDP52 | NM_005079 | miR-96, miR-182 | Forward: GCAAAGGTAGAAGAAGAAATCCA  Reverse: ACCCTTTGGCAATGTTCTGT |
| CACNA2D2 | NM_001005505 | miR-96, miR-182 | Forward: TCAAGGAGGAAGACATCGTG  Reverse: GTTTGGGTCCTCGATGAAGT |
| LRP1B | NM_018557 | miR-183 | Forward: AGATGGAAGCGATGAGCTTT  Reverse: CGTCATCATGGTCACAAACA |

^1^List of putative conserved miR-183/96/182 targets and their primers in breast cancer: The first column represents the official symbol of the candidate target; the second column is the RefSeq accession number; the third column indicates which members of the miR-183/96/182 cluster are predicted to target each mRNA; the fourth column represents the sequences of primer sets for real time PCR for each gene.

**Table S3. Primer sets for pri-miRNA transcription screening^1^**

| **Name** | **Forward** | **Reverse** | **Location** |
| --- | --- | --- | --- |
| Seq#1 | CAACCGGAGCCACATTCAAG | CACCCGTAGAGCTGAGTGAC | -5352bp to -3737bp |
| Seq#2 | GCCATTCGTTTGCTCTTCCA | CCTAACCCAAATGCGACAGG | -3991bp to -2370bp |
| Seq#3 | AGCTCCTCTTACCCGCTTTT | CTGGGAAGGTTGCATCAGTG | -2522bp to -821bp |
| Seq#4 | ATTTTCTAGCCACCCTGCCT | GGATGGCCCTACAGATGGTT | -1058bp to 584bp |
| Seq#5 | AGATCGCCTTACACTGCCTG | GGCTCCAGGGAAAAGCTCTA | 463bp to 2073bp |
| Seq#6 | TGAGTGAGAATGAGCCCTGG | GTTCCCACTTCCCCTGATGA | 1972bp to 3621bp |
| Seq#7 | TCCCATCTCACTCCACCCTA | CTTCCCAGCTGACTTGAGGA | 3303bp to 4811bp |
| Seq#8 | CTAGGGATGGTGTCTGCTCC | CGGGAGAACAGTTCCCTTCT | 4257bp to 5893bp |

^1^Primer sets for pri-miRNA transcription screening were listed in order: The first column is the name for the piece of DNA; the second and third columns represent the sequences of forward and reverse primers for RT-PCR; the fourth column indicates the location of this piece of DNA calculated from the start site of miR-183 pre-microRNA.

**Table S4. The correlations between miRNAs’ targets/regulators and surface markers^1^**

|  | RAB21 | HSF2 | ZEB1 |
| --- | --- | --- | --- |
| ER- **(n=**112**)** | -0.036±0.570 | -0.390±0.663 | -0.234±1.010 |
| ER+ **(n=**389**)** | 0.284±0.507^***^ | -0.714±0.467^***^ | 0.283±0.968^***^ |
| PR- **(n=**169**)** | 0.114±0.577 | -0.465±0.634 | -0.086±0.998 |
| PR+ **(n=**331**)** | 0.264±0.512^**^ | -0.730±0.456^***^ | 0.299±0.979^***^ |
| HER2- **(n=**271**)** | 0.236±0.507 | -0.643±0.540 | 0.194±0.993 |
| HER2+ **(n=**90**)** | 0.235±0.566 | -0.582±0.617 | 0.321±0.942 |

^1^The expressions of miRNAs’ targets/regulators in different breast cancer subtypes based on their surface markers: Patient number is indicated in the first column. Data are shown in means±S.D. Expression levels are log2 lowess normalized (cy5/cy3). Statistical probability (*p*) was expressed as **p*<0.05, ***p*<0.01, ****p*<0.001.

**Table S5. miRNAs’ targets/regulators in different molecular subtypes of breast cancer^1^**

|  | RAB21 | HSF2 | ZEB1 |
| --- | --- | --- | --- |
| **HER2 enriched (n=24)** | -0.115±0.528^**^ | -0.454±0.740 | 0.116±0.915 |
| **Basal (n=53)** | 0.009±0.572^**^ | -0.250±0.722^***^ | -0.493±1.010^***^ |
| **Luminal A (n=217)** | 0.292±0.477 | -0.740±0.438^***^ | 0.366±0.916^***^ |
| **Luminal B (n=66)** | 0.343±0.533 | -0.628±0.566 | 0.374±0.973 |

^1^The expressions of miRNAs’ targets/regulators in different molecular subtypes of breast cancer: Patient number is indicated in the first column. Data are shown in means±S.D. Expression levels are log2 lowess normalized (cy5/cy3).The following markers were used to determine breast cancer subtypes: luminal A (ER+ and/or PR+, HER2-), luminal B (ER+ and/or PR+, HER2+), basal-like (ER-, PR- , HER2-), HER2 enriched (ER-, PR-, HER2+). Statistical probability (*p*) was expressed as **p*<0.05, ***p*<0.01, ****p*<0.001.
