# Supplementary material for: MiR-183/-96/-182 cluster is up-regulated in most breast cancers and increases cell proliferation and migration
Source: Breast Cancer Res. 2014 Nov 14;16:473. doi: 10.1186/s13058-014-0473-z (PMC4303194; doi:10.1186/s13058-014-0473-z)
Supplement: Supplementary file 7 — Additional file 7: Figure S4.: Test of the miRNA knockdown effects in basal-like breast cancer cells. (PDF 4 MB) [file 13058_2014_473_MOESM7_ESM.pdf]

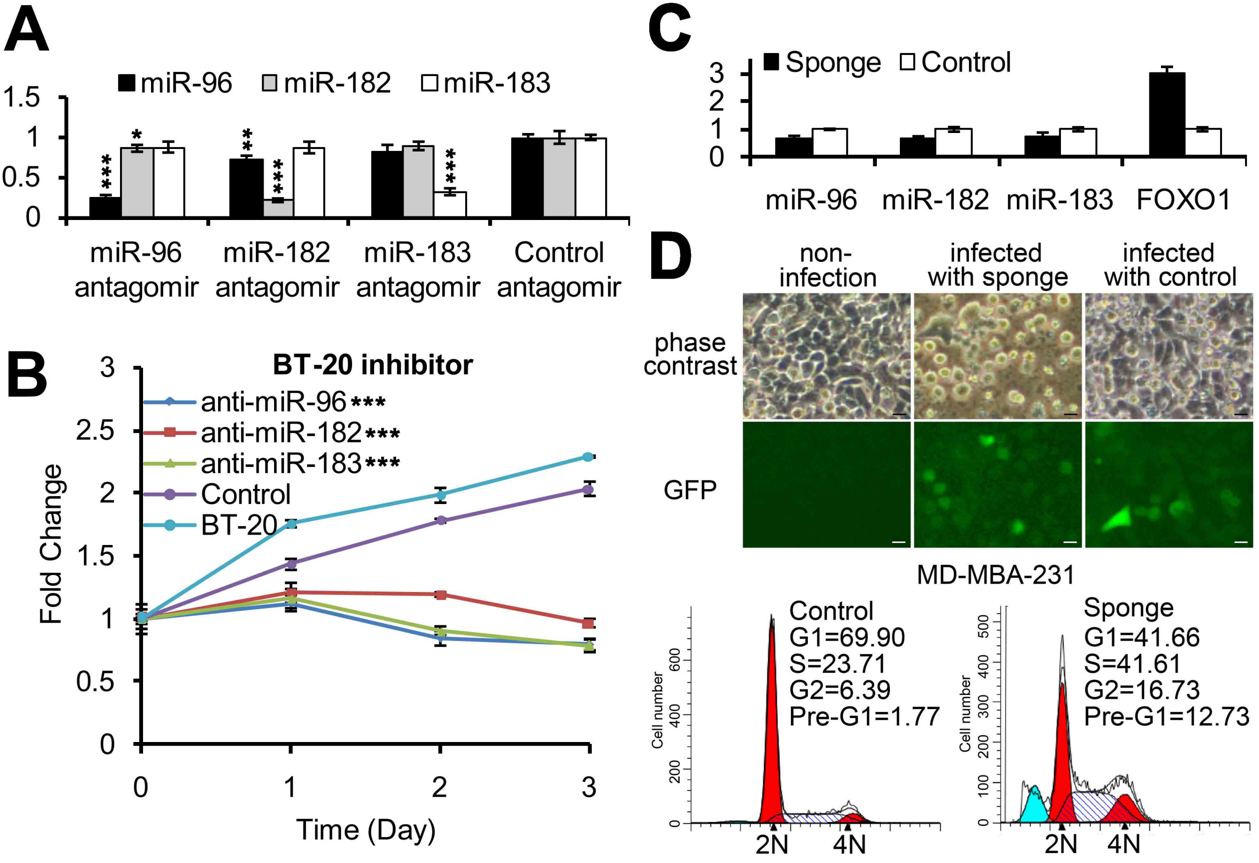

Figure S4. Test of the miRNA knockdown effects in basal like breast cancer cells. (A) Real-time PCR results showed the knockdown efficiency and specificity of miR-DownTM antagomirs in BT-20 cells. U6 snRNA was used as internal control. Error bars represent standard deviation (n = 4). **(B)** MTT assays showed the cell growth rates of BT-20 cells after knockdown of *miR-183/-96/-182* cluster. Error bars represent standard deviation (n = 4). **(C)** Test of the inhibition efficiency of *miR-183/-96/-182* cluster sponge lentivirus by Real-time PCR. Error bars represent standard deviation (n = 3). **(D)** MDA-MB-231 cells infected with *miR-183/-96/-182* cluster sponge lentivirus underwent apoptosis 3 days after transduction. Scale bars: 20 µm. The upper panels showed the phase contrast and green fluorescent micrographs of indicated cells. The bottom panels showed the flow cytometric graph of indicated cells.
