# Supplementary material for: MiR-183/-96/-182 cluster is up-regulated in most breast cancers and increases cell proliferation and migration
Source: Breast Cancer Res. 2014 Nov 14;16:473. doi: 10.1186/s13058-014-0473-z (PMC4303194; doi:10.1186/s13058-014-0473-z)
Supplement: Supplementary file 8 — Additional file 8: Figure S5.: Inhibition two or three of the cluster members at one time induced apoptosis in T47D cells. (PDF 1 MB) [file 13058_2014_473_MOESM8_ESM.pdf]

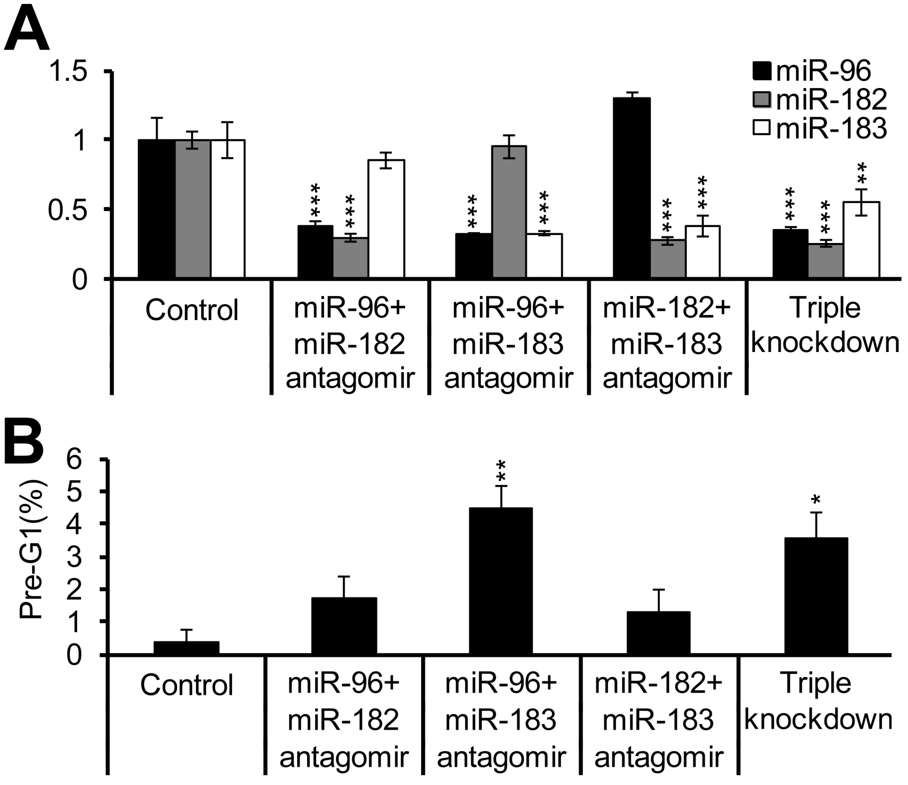

Figure S5. Inhibition two or three of the cluster members at one time induced apoptosis in T47D cells. (A) Real time PCR results showed the knockdown efficiency and specificity of miR-Down™ antagomirs. **(B)** Flow cytometric analysis showed the pre-G1 cell percentages were increased in cells treated with 2 or 3 antagomirs.
